# Monocyte-Derived LGMN^+^ Macrophages Divert Lung Injury Outcomes toward Fibrosis through Matrix Remodeling

**DOI:** 10.34133/research.1341

**Published:** 2026-06-29

**Authors:** Zhongzheng Li, Yujie Zhang, Kun Yang, Diwen Zheng, Yulong Chen, Yuqing Zhang, Xin Pan, Peishuo Yan, Ivan O. Rosas, Guoying Yu, Lan Wang

**Affiliations:** ^1^ College of Life Science, Henan Normal University, Xinxiang, Henan 453007, China.; ^2^ Department of Pharmacy, The First Affiliated Hospital of Zhengzhou University, Zhengzhou, Henan 450002, China.; ^3^ Division of Pulmonary, Critical Care and Sleep Medicine, Baylor College of Medicine, Houston, TX 77030, USA.

## Abstract

Pulmonary fibrosis (PF) is a fatal interstitial lung disease characterized by excessive extracellular matrix deposition and irreversible architectural distortion. The mechanisms driving the transition from tissue repair to fibrosis are complex and remain poorly understood. By analyzing interpatient variation across 75 idiopathic PF lungs, we identified a conserved profibrotic macrophage subset, distinct from canonical M1/M2 or SPP1^+^ states, characterized by high legumain (*LGMN*) expression and enrichment of gene signatures implicated in leukocyte activation and matrix remodeling. *LGMN*^+^ macrophages localize within fibroblastic foci and are associated with disease progression and poor prognosis. Lineage-tracing and RNA velocity analyses revealed that *LGMN*^+^ macrophages arise from monocytes through fibroblast-derived macrophage colony-stimulating factor signaling, which activates Maf BZIP transcription factor B-dependent differentiation programs. Pharmacological inhibition or macrophage-specific deletion of *Lgmn* markedly attenuated bleomycin-induced lung fibrosis, reduced extracellular matrix accumulation, and improved lung architecture. LGMN activates cathepsin S to mediate degradation of basement membrane collagen IV, thereby disrupting the alveolar–capillary barrier. In parallel, secreted LGMN acts as a paracrine signal to activate fibroblasts and promote collagen I deposition, collectively fostering a profibrotic niche. Together, these findings establish LGMN as a macrophage effector that links immune activation to matrix remodeling, thereby driving the transition from tissue injury to fibrosis.

## Introduction

The capacity for scarless regeneration in immature tissues, demonstrated in murine and human embryos and fetuses prior to inflammatory onset, points to inflammation as a potential trigger of fibrosis [1,2]. Acute inflammation resolves once the injurious stimulus is eliminated and tissue architecture is restored [3]. However, persistent underlying injury drives the transition from acute to chronic inflammation, accompanied by a shift from short-lived neutrophils to longer-lived lymphocytes and macrophages [4,5]. Persistent inflammation of this kind can ultimately culminate in fibrotic tissue remodeling [5]. In the context of the lung, chronic inflammation triggered by recurrent microinjuries promotes aberrant repair and excessive extracellular matrix (ECM) deposition, ultimately leading to pulmonary fibrosis (PF)—the terminal stage of various chronic interstitial lung diseases [1,6,7]. Despite decades of investigation, the precise roles of monocytes and macrophages in PF pathogenesis remain contentious [8,9]. While early studies highlighted their potential importance, the limited clinical benefit of corticosteroid therapy in idiopathic pulmonary fibrosis (IPF) challenged this view and prompted a critical reassessment of their pathogenic contribution [10,11].

The limited efficacy of anti-inflammatory therapies in IPF led researchers to propose that inflammation might play a less central role in disease pathogenesis than previously assumed [12,13]. This notion challenged the classical view that fibrosis is merely a downstream consequence of inflammation and shifted focus toward macrophage-intrinsic mechanisms in fibrogenesis [14]. Recent studies have further underscored the contribution of the self-function of monocytes and macrophages to fibrotic progression [15,16]. For example, macrophages participate actively in aberrant tissue remodeling by releasing a spectrum of profibrotic mediators—including transforming growth factor-β1 (TGF-β1), C-C motif chemokine ligand 18 (CCL18), galectins, connective tissue growth factor (CTGF), and matrix metalloproteinases—that promote fibroblast migration, proliferation, activation, and differentiation into myofibroblasts [17–20]. Notably, corticosteroids and other anti-inflammatory agents fail to suppress and may even boost profibrotic mediator release by alveolar macrophages (AMs) ex vivo, underscoring their inflammation-independent role in fibrosis [21,22]. Experimental depletion of lung macrophages during the fibrotic phase markedly attenuates PF, reinforcing their role as key effectors and potential therapeutic targets [15]. Nevertheless, the absence of precise molecular definition of fibrosis-associated macrophage subsets has hindered the development of macrophage-directed interventions.

Over the past 3 decades, the classification of macrophages has undergone multiple paradigm shifts. The earliest system, established between 1990 and 2010, defined macrophages as classically activated (M1) or alternatively activated (M2) subsets [16,23]. Although this framework provided a convenient in vitro model, it merely reflected macrophage responses to external stimuli and failed to represent their true in vivo states [8,19]. To better capture macrophage diversity under physiological conditions, subsequent classification systems incorporated tissue localization and ontogeny, distinguishing AMs, monocyte-derived AMs, and interstitial macrophages [16,24,25]. However, in the severely disrupted architecture of fibrotic lungs, this structure-based classification collapses, as spatial boundaries between macrophage niches become indistinguishable. The advent of single-cell RNA sequencing (scRNA-seq) in recent years has further expanded our understanding of macrophage heterogeneity, leading to the identification of several fibrosis-associated subpopulations, such as *SPP1*^+^, *CHIT1*^+^, and *Cx3cr1*^+^*SiglecF*^+^ macrophages [26–30]. Nevertheless, these approaches primarily rely on transcriptional markers without integrating functional interpretation, limiting their translational potential. For instance, the recently proposed targeting of *SPP1*^+^ macrophages depends on TGF-β inhibition—an intervention with poor clinical feasibility [29,31,32]. Moreover, the foundational assumption that *SPP1*^+^ macrophages represent a pathogenic population distinct from normal macrophages is now being challenged. Emerging data suggest that *SPP1* expression may reflect a downstream consequence of tissue injury rather than a causal driver of fibrosis [33,34].

These ongoing uncertainties expose 2 critical gaps. First, there is a need to identify macrophage populations that track with disease progression rather than the difference between normal and static disease states. Second, functional insights must accompany transcriptional definitions to enable mechanistic understanding and therapeutic translation. To address these challenges, we integrated scRNA-seq data from 75 IPF lung samples encompassing 278,187 cells. This analysis uncovered a distinct macrophage subset characterized by high *LGMN* expression, which is independent of classical M1/M2 or *SPP1*^+^ classifications and tightly associated with disease progression. Both pharmacological inhibition and macrophage-specific deletion of LGMN markedly attenuated PF. Mechanistically, spatial transcriptomic analysis revealed colocalization of *LGMN*^+^ macrophages with fibroblast foci in human fibrotic lungs. *LGMN*^+^ macrophages arise from monocytes stimulated by fibroblast-secreted macrophage colony-stimulating factor (M-CSF), which activates Maf BZIP transcription factor B (MAFB)-dependent differentiation programs. Functionally, *LGMN*^+^ macrophages exhibit strong activity in leukocyte migration and ECM remodeling, and drive fibrotic remodeling through the LGMN–CTSS axis. LGMN promotes fibrosis by activating CTSS, which collapses type IV collagen and disrupts the alveolar–capillary structure to contribute space for type I collagen deposition. This work not only redefines the landscape of macrophage heterogeneity in fibrosis but also reveals LGMN as a promising therapeutic target for halting disease progression.

## Results

### Interpatient heterogeneity in IPF reveals macrophage signatures linked to disease progression and clinical outcomes

**Fig. 1.**
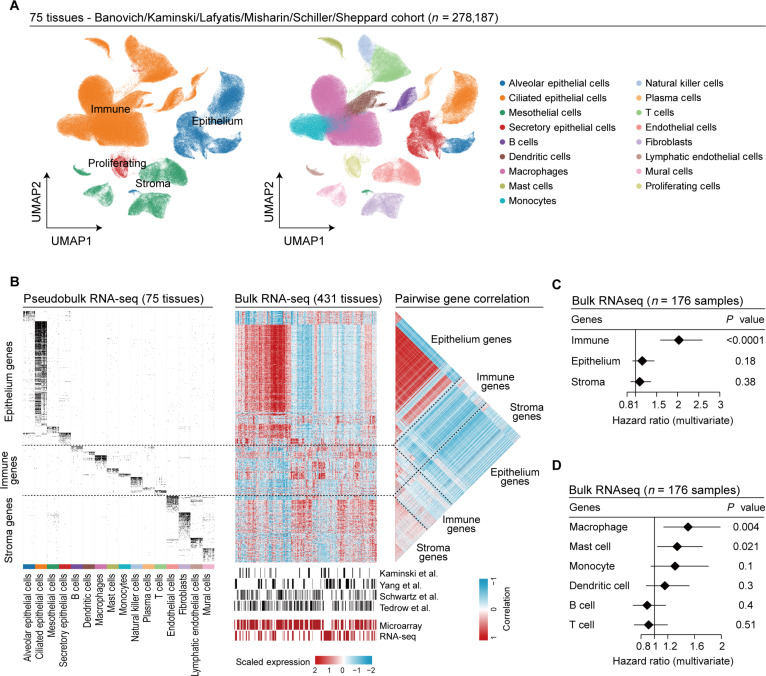
Macrophages is a major contributor to the clinical outcome of IPF. (A) Uniform manifold approximation and projection (UMAP) visualization of single-cell transcriptomic profiles combined from all 75 samples from the 6 cohorts, highlighting the separation of main cell lineages and showing finer clustering within each. (B) (Left) Identification of genes that are dominantly expressed (with fold change > 3 relative to the next-most-expressing cell type; multiple-test testing *P* value < 0.05 for patient-level comparison) in each major cell type and based on scRNA-seq data from the 6 cohorts. (Middle) Projection of these genes to bulk RNA-seq data from public datasets (totaling *n* = 431 IPF patients) shows their relative expression pattern together with associated cohort of origin. (Right) Pairwise correlations between the same genes. The patients were sorted according to hierarchical clustering. (C) Comparison of 3 prognostic signatures, each derived using epithelial, stromal, and immune genes only. Multivariable Cox regression was used to obtain the hazard ratios (with Wald 95% confidence intervals shown as horizontal bars, and *P* values given on the right) based on the cross-validated prognostic scores derived using the GLMNET Cox model and applied to pairwise differences of expression of the genes. (D) Similar analysis as in (C), but using genes dominant in each subdivision of the immune cell types.

We reanalyzed the public scRNA-seq data from 75 lung tissue samples in IPF, without prior selection or enrichment for any specific cell type. To minimize dataset-specific batch effects, we evaluated 51 published integration algorithms on 6 independent datasets using previously established benchmarking pipeline [35]. Harmony was selected to generate an integrated IPF atlas comprising 278,187 cells (Fig. S1A). The atlas encompassed 3 major cellular compartments of the fibrotic lung, including epithelium, stroma, and immune. We also captured all major cell types, namely, epithelial cells, fibroblasts, endothelial cells, lymphocytes, myeloid cells, etc. (Fig. 1A). Cluster-specific genes were used to annotate cell types with classic markers described in previous studies (Fig. S1B). We next identified 1,563 compartment-dominant genes, defined as those expressed at least threefold higher in a given compartment relative to all others (Fig. 1B, left). These genes were subsequently used to deconvolute bulk transcriptomic data from 431 IPF patients pooled from 4 independent cohorts. The expression heatmap of these genes in all 431 patients revealed extensive intercohort mixing, confirming the overall homogeneity of transcriptional profiles (Fig. 1B, middle). Finally, pairwise correlation analysis showed that the fibrotic microenvironment compartment dominant genes were coenriched in their respective cellular compartments, indicating that what becomes apparent as coregulation in bulk mRNA data may in fact largely be driven by heterogeneity in abundance of various fibrotic microenvironment cell types (Fig. 1B, right). Notably, stromal cell transcriptomes were more closely associated with those of immune cells than with those of epithelial cells, suggesting an orchestrated communication between stromal and immune compartments (Fig. 1B, right). To determine which cellular compartments were most closely linked to clinical outcome, we used supervised modeling approaches commonly applied to bulk RNA-seq data that include clinical information, but restricted the analysis to cell type-dominant genes as feature variables. Gene expression values were internally normalized using a pairwise log-ratio transformation, and multipair signatures were generated via an elastic-net classifier trained on overall survival. In univariate analyses, both epithelial and immune cell compartment showed significant prognostic associations (Fig. S1C). However, in a multivariate model including all 3 compartments (epithelial, stromal, and immune), only the immune signature retained independent prognostic value (Fig. 1C), implicating immune cell compartment as a key determinant of disease progression. Given the functional diversity of immune cell subsets, we next assessed their individual prognostic contributions. Univariate analysis indicated that nearly all major immune cell types were significantly associated with outcome (Fig. S1D), yet multivariate analysis revealed that only mast cells (*P* = 0.021) and macrophages (*P* = 0.004) provided nonredundant prognostic information (Fig. 1D). Kaplan–Meier survival analyses further supported the strong prognostic relevance of macrophage-specific signatures (Fig. S1E). In summary, we highlight macrophages as central mediators of the fibrotic phenotype and key determinants of patient outcomes.

### *LGMN*^+^ macrophages are identified as a conserved fibrosis-associated population in humans and mice

**Fig. 2.**
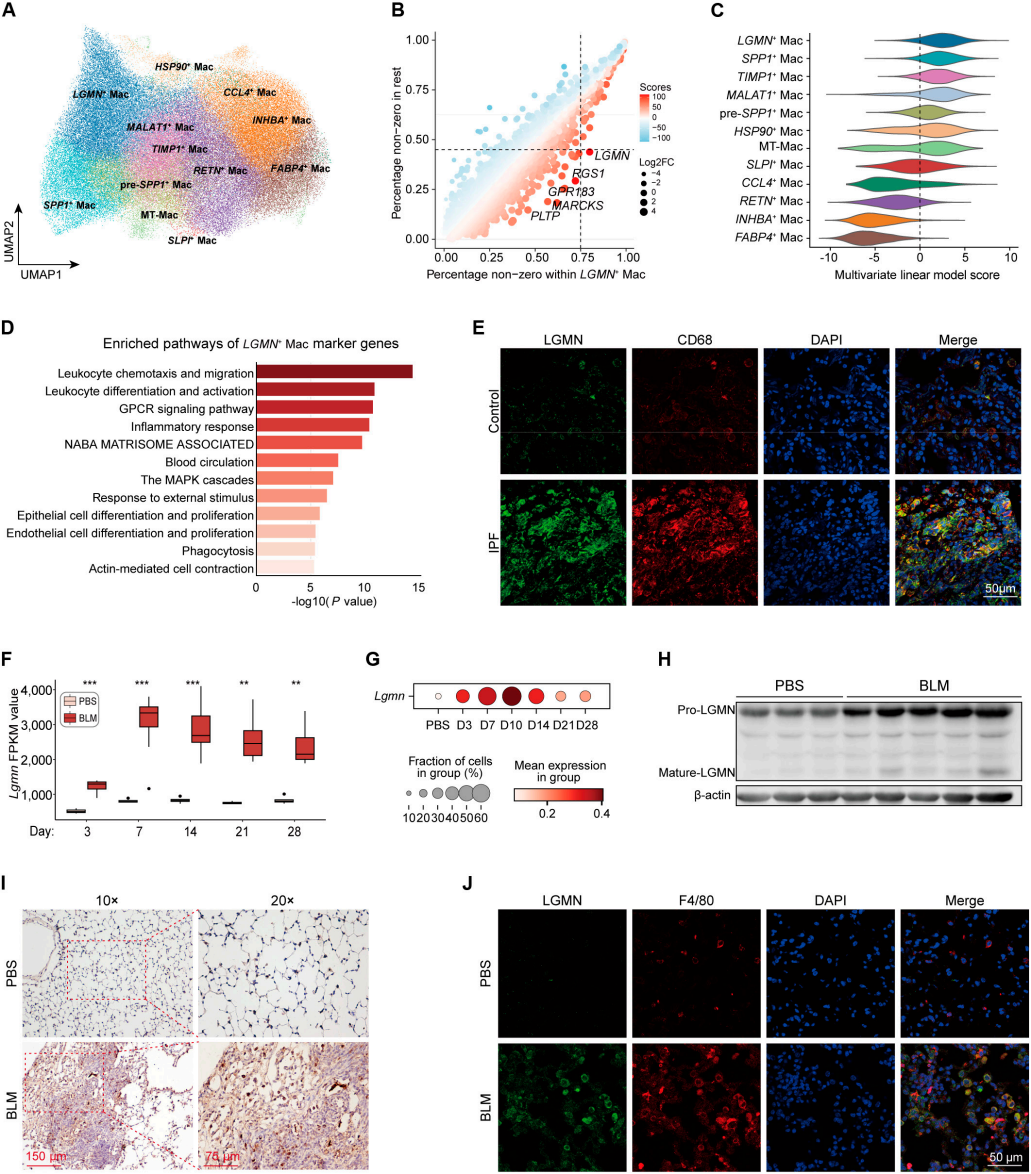
*LGMN*^+^ macrophages represent a conserved fibrosis-associated macrophage population in humans and mice. (A) UMAP plot of lung macrophages from IPF, showing distinct macrophage subsets defined by marker gene expression. (B) Expression specificity of genes in *LGMN*^+^ Mac compared to resting cells. Points represent genes, colored by log_2_FC and sized by expression score. Enriched genes (e.g., *RGS1* and *GPR183*) show higher expression in *LGMN*^+^ Mac. (C) Violin plots show the distribution of predicted scores based on gene expression, with positive scores indicating high prognostic risk and negative scores denoting low risk. Each point represents a macrophage subset (e.g., *LGMN*^+^ Mac and *SPP1*^+^ Mac). The central line marks the median, and the width reflects data density. (D) Enrichment analysis (GSEA) of hallmark pathways enriched in LGMN+ macrophage marker genes, highlighting roles in leukocyte chemotaxis, migration, differentiation, and ECM remodeling. (E) Immunofluorescence staining of lung tissue sections from control and IPF showing colocalization of LGMN (green) and CD68 (red), with nuclei stained by DAPI (blue). Scale bar: 50 μm. (F) RNA-seq analysis of Lgmn mRNA expression over time post-BLM administration. Data shown as mean ± SEM; **P < 0.01, ***P < 0.001. (G) Dot plot of Lgmn expression across lung macrophage subsets identified by scRNA-seq. Color denotes mean normalized expression, and dot size corresponds to the proportion of expressing cells in each cluster. (H) Western blot analysis of pro-LGMN and mature LGMN protein levels in lung lysates from PBS- and BLM-treated mice. β-actin serves as loading control. (I) Immunohistochemistry staining of lung sections at 10× and 20× magnification showing increased cellular infiltration and fibrotic changes in BLM-treated mice. Scale bars: 150 μm (10×) and 75 μm (20×). (J) Immunofluorescence staining of LGMN (green) and F4/80 (red) in lung tissues from PBS- and BLM-treated mice, with DAPI (blue) marking nuclei. Merged images show colocalization of LGMN+ macrophages in fibrotic lesions. Scale bar: 50 μm.

To delineate macrophage populations associated with PF, we reintegrated 167,856 macrophage from the generated IPF atlas and generated Leiden clustering, which resolved 12 transcriptionally distinct macrophage subsets (Fig. 2A and Fig. S2A). Among these, macrophages with high *LGMN* expression exhibited the strongest prognostic association and were thus designated as fibrosis-associated macrophages (Fig. 2B and C). Pathway enrichment analysis of *LGMN*^+^ marker genes revealed strong activation of programs involved in leukocyte migration and ECM remodeling, consistent with a profibrotic phenotype (Fig. 2D). Macrophage activation has traditionally been classified into proinflammatory (M1) and profibrotic (M2) states, defined by in vitro stimulation with lipopolysaccharide (LPS)/interferon-gamma (IFN-γ) or interleukin-4 (IL-4), respectively. M2-like phenotype has long been implicated in fibrosis progression. Notably, *LGMN*^+^ macrophages lacked enrichment for canonical M1 or M2 gene signatures, indicating that they represent a transcriptionally distinct macrophage state beyond the conventional polarization paradigm (Fig. S2B). Diffusion-map trajectory analysis identified 3 terminal macrophage fates—*LGMN*^+^, *SPP1*^+^, and *FABP4*^+^—with the *LGMN*^+^ population forming an independent lineage, distinct from the previously reported profibrotic *SPP1*^+^ subset (Fig. S2C). Pseudo-bulk RNA-seq analysis reveals that *LGMN*^+^ and *SPP1*^+^ macrophages exhibit markedly distinct transcriptional signatures: *LGMN*^+^ macrophages are uniquely enriched for genes such as *FCGR3A, MS4A6A, C1QA*, and *TGFBI*, whereas *SPP1*^+^ macrophages prominently express *MATK, LPL, CD52*, and *GPC4* (Fig. S2D). Specifically, *SPP1*^+^ macrophages show significant enrichment in pathways related to oxidative phosphorylation and DNA repair. In contrast, *LGMN*^+^ macrophages are strongly enriched for hallmark signatures of hypoxia, and inflammatory response, consistent with a role in sensing and amplifying tissue injury under low-oxygen and proinflammatory conditions (Fig. S2E).

At the molecular level, both LGMN mRNA and protein levels were markedly up-regulated in human fibrotic lungs (Fig. S2F and G). IPF patients with higher *LGMN* expression exhibited significantly worse clinical outcomes, whereas expression of classical M1 (*CD86* and *CD80*) or M2 (*MSR1* and *MRC1*) markers showed no correlation with disease severity (Fig. S2H). Univariate Cox regression analysis revealed that high LGMN expression was significantly associated with worse survival (HR > 1, *P* < 0.0001, Fig. S2I). Importantly, in the multivariate model adjusting for age and sex, LGMN remained a strong independent prognostic factor (*P* < 0.0001, Fig. S2J). These results collectively establish LGMN as a robust and independent predictor of adverse clinical outcome. Public spatial transcriptomic demonstrated that LGMN expression was concentrated within fibrotic foci (Fig. S2K), and we confirmed via immunofluorescence that LGMN localizes to macrophages within IPF fibrotic lesions (Fig. 2E). Cross-species analysis using the UniProt database revealed that LGMN is highly conserved, supporting its functional study in murine models. In both bleomycin (BLM)- and TGF-β-induced fibrosis models, *Lgmn* transcripts were markedly elevated (Fig. 2F and Fig. S2L). Analysis of public scRNA-seq data across time courses of BLM-induced fibrosis showed a peak in *Lgmn*-expressing macrophages on day 10 postchallenge (Fig. 2G). Correspondingly, LGMN protein levels were substantially increased in fibrotic mouse lungs (Fig. 2H and Fig. S2M). Immunohistochemistry confirmed enrichment of LGMN within macrophages residing in mouse lung fibrotic region (Fig. 2I), and coimmunofluorescence validated its colocalization with the macrophage marker F4/80, both markedly enhanced upon fibrosis induction (Fig. 2J). Collectively, these findings identify *LGMN*^+^ macrophages as a transcriptionally distinct, evolutionarily conserved, and spatially localized profibrotic subset that transcends classical M1/M2 paradigms.

### Fibroblast-derived M-CSF drives the differentiation of monocytes into *LGMN*^+^ macrophages through activation of MAFB

**Fig. 3.**
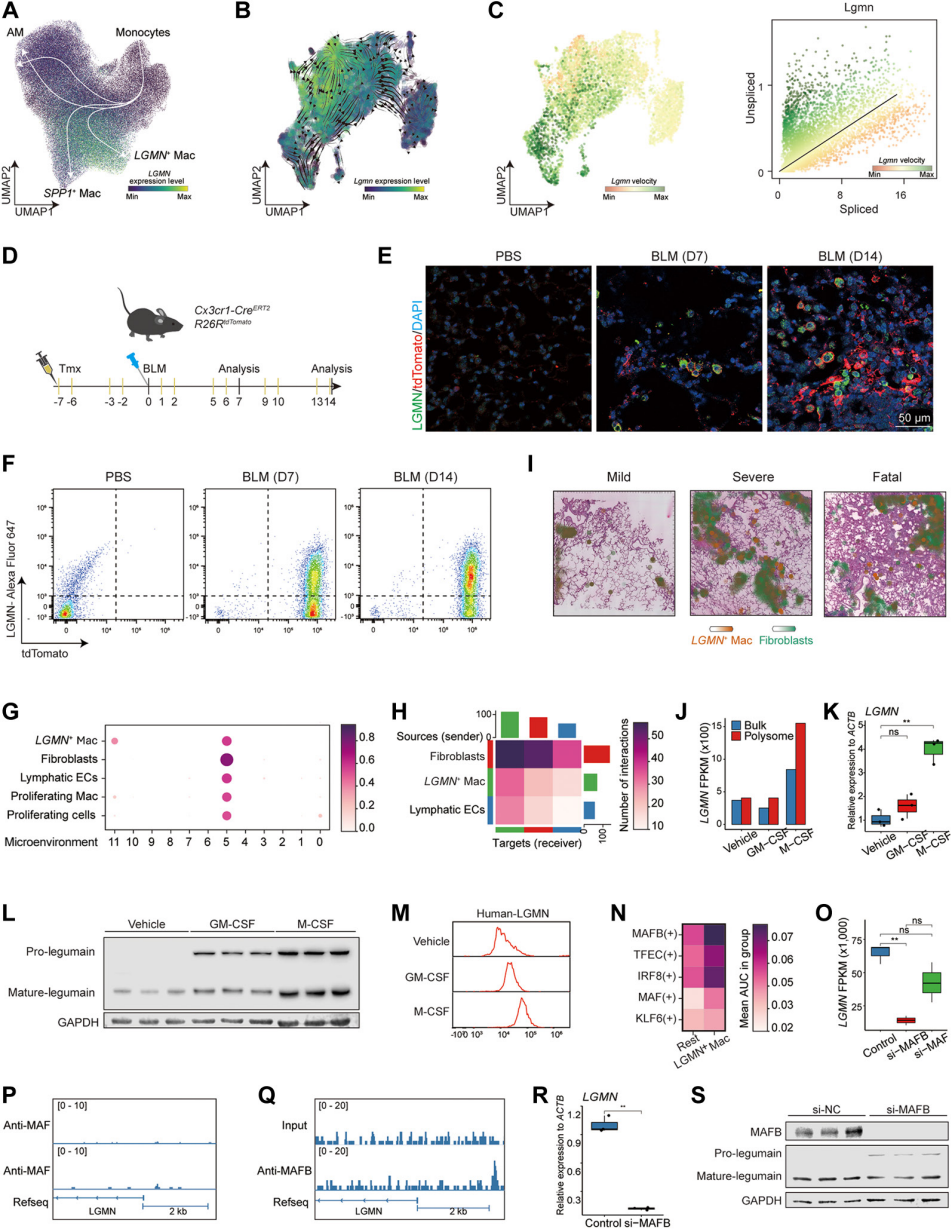
Fibroblast-derived M-CSF drives the differentiation of monocytes into *LGMN*^+^ macrophages through activation of MAFB. (A) UMAP representation of human lung myeloid cells from patients with pulmonary fibrosis, colored by their differentiation trajectory as predicted by Slingshot. Three major trajectories are identified. (B) RNA velocity analysis reveals a developmental continuum from monocyte precursors to mature *LGMN*^+^ Mac, with increasing *Lgmn* expression along the differentiation path. (C) RNA velocity of *Lgmn*. Left: UMAP colored by *Lgmn* velocity. Right: Spliced vs. unspliced transcript levels, with color indicating velocity magnitude. Cells above the diagonal are transitioning toward higher *Lgmn* expression. (D) Experimental design: *Cx3cr1*-Cre^ERT2^; R26R^tdTomato^ mice were administered tamoxifen (Tmx) prior to BLM challenge. Lung tissues were analyzed on days 5, 7, 9, 10, 13, and 14 post-BLM to track macrophage lineage dynamics. (E) Immunofluorescence staining show temporal expansion of *LGMN*^+^ Mac (red) within fibrotic foci, colocalizing with fibroblasts and collagen deposition. Arrowheads indicate representative clusters of *LGMN*^+^ Mac infiltrating fibrotic lesions. (F) Flow cytometry plots of tdTomato^+^ cells (derived from monocytes) gated on LGMN-Alexa Fluor 647 expression over time. (G) Cell–cell communication network analysis identifies LGMN^+^ Mac as major signal sources in the fibrotic microenvironment, particularly interacting with fibroblasts, lymphatic endothelial cells (ECs), and proliferating macrophages. (H) Interaction heatmap showing ligand–receptor pairs between *LGMN*^+^ Mac and surrounding stromal and immune cells, highlighting key pathways involved in fibroblast. (I) Histological comparison of lung sections across disease severity (mild, severe, and fatal). *LGMN*^+^ Mac (orange) accumulate progressively in fibrotic areas, correlating with worsening pathology. Fibroblasts are shown in green. (J) Bulk and polysomal RNA sequencing reveal increased *Lgmn* mRNA level in M-CSF-treated macrophages compared to vehicle or GM-CSF controls, suggesting translational regulation by cytokine milieu. (K) Quantification of *Lgmn* expression in human PBMC-derived monocytes treated with GM-CSF or M-CSF. GM-CSF significantly enhances *Lgmn* expression (*n* = 3 biological replicates; **P < 0.01). (L) Western blot analysis of pro- and mature LGMN protein level in murine macrophages after treatment with GM-CSF or M-CSF. (M) Flow cytometry of human LGMN expression. Macrophages treated with vehicle, GM-CSF, or M-CSF show differential LGMN expression. (N) Enrichment analysis of transcription factor binding motifs in the *Lgmn* promoter region. (O) RNA-seq quantification of *Lgmn* expression in MDMs transfected with si-*MAFB* vs. control siRNA. *MAFB* knockdown significantly reduces Lgmn expression (*n* = 3; **P < 0.01). (P) ChIP-seq tracks showing enrichment of MAFB binding at the Lgmn locus in anti-MAF experiments. (Q) Input and control ChIP-seq signals confirm specificity of MAFB binding near the Lgmn gene. (R) qRT-PCR validation of Lgmn knockdown in macrophages transduced with si-*MAFB* versus control siRNA. Expression is significantly reduced (*n* = 3; **P < 0.01). (S) Western blot confirming reduction of both pro- and mature LGMN protein level upon MAFB silencing. β-actin used as loading control.

To determine the developmental origin of *LGMN*^+^ macrophages, we integrated monocytes into the established macrophage atlas and performed pseudotime trajectory analysis. This analysis revealed 4 differentiation trajectories originating from monocytes, leading to 2 AM fates and 2 distinct fates (*SPP1*^+^ and *LGMN*^+^ macrophages) (Fig. 3B). To further validate the differentiation trajectories of *LGMN*^+^ macrophages in mice, we reanalyzed a previously published scRNA-seq dataset of murine immune cells [36]. RNA velocity analysis confirmed that *LGMN*^+^ macrophages were transcriptionally downstream of monocytes (Fig. 3B). Examination of *LGMN* RNA velocity revealed that unspliced *LGMN* transcripts were abundant in monocytes, but not in AMs, suggesting that *LGMN*^+^ macrophages arise from monocyte differentiation rather than transdifferentiation of resident AMs (Fig. 3C). To experimentally validate this inference, we performed lineage tracing of monocytes in vivo (Fig. 3D). Flow cytometry and immunofluorescence staining demonstrated that *Lgmn*^+^ macrophages were nearly absent on day 0, but markedly expanded on days 7 to 14 after BLM treatment, supporting a monocyte-derived origin of *Lgmn*^+^ macrophages (Fig. 3E and F).

We next investigated the microenvironmental cues driving monocyte differentiation toward *LGMN*^+^ macrophages. Spatial niche analysis revealed that *LGMN*^+^ macrophages, fibroblasts, and lymphatic endothelial cells were enriched within the same fibrotic niche (Fig. 3G). CellChat analysis predicted strong ligand–receptor interactions between fibroblasts and macrophages (Fig. 3H). In sections with different degrees of fibrosis, through deconvolution, it was also observed that *LGMN*^+^ macrophages and fibroblasts were coenriched in the fibrotic lesions (Fig. 3I). Specifically, fibroblast-derived M-CSF (CSF1) and macrophage-expressed CSF1R formed a dominant signaling axis (Fig. S3A). Public transcriptomic datasets further confirmed that M-CSF, but not granulocyte-macrophage colony-stimulating factor (GM-CSF), induced robust up-regulation of LGMN at both mRNA level and protein translation rate in peripheral blood mononuclear cell (PBMC)-derived monocytes (Fig. 3J). Consistently, Western blotting, real-time quantitative PCR (qPCR), and flow cytometry analyses verified that M-CSF—but not GM-CSF—enhanced LGMN expression in human PBMC- and mouse bone marrow-derived monocytes (Fig. 3K to M and Fig. S3B to D).

We then asked how M-CSF promotes *LGMN* expression. SCENIC analysis identified significant activation of the MAF family transcription factors MAF and MAFB in *LGMN*^+^ macrophages (Fig. 3N). Supporting this, previously published RNA-seq data showed that knockdown of MAFB down-regulated LGMN expression (Fig. 3O). Analysis of chromatin immunoprecipitation sequencing (ChIP-seq) datasets from M-CSF-stimulated human PBMC-derived monocytes revealed a prominent MAFB binding peak ~2 kb upstream of the LGMN locus, whereas no such enrichment was observed for MAF (Fig. 3P and Q). siRNA-mediated MAFB knockdown in both PBMC-derived macrophages and bone marrow-derived macrophages (BMDMs) significantly decreased LGMN mRNA and protein levels (Fig. 3R and S and Fig. S3E and F), confirming that MAFB directly regulates *LGMN* transcription downstream of M-CSF signaling. These findings suggested that *LGMN*^+^ macrophages originate from monocytes, not resident AMs, in both mouse and human lungs, where they are driven by fibroblast-derived M-CSF signaling through CSF1R to activate the transcription factor MAFB, which directly up-regulates LGMN expression.

### Pharmacological inhibition and macrophage-specific genetic ablation of LGMN attenuate BLM-induced PF

**Fig. 4.**
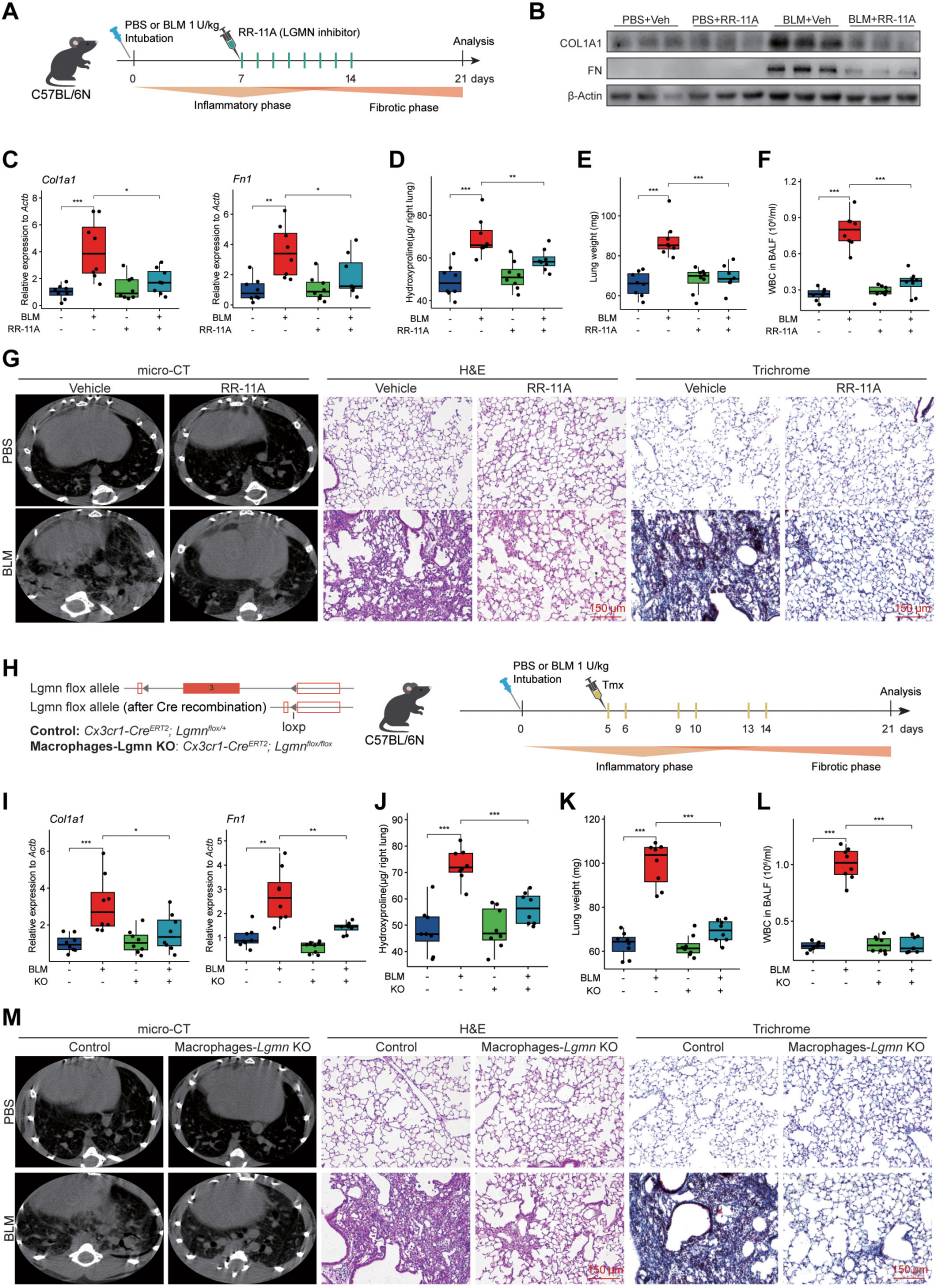
Pharmacological inhibition and macrophage-specific genetic ablation of LGMN attenuate BLM-induced PF. (A) Experimental timeline showing intratracheal administration of PBS or BLM followed by treatment with RR-11A or vehicle during the inflammatory and fibrotic phases of lung injury. Mice were analyzed on day 21 post-BLM. (B) Representative Western blot analysis of COL1A1 and FN protein levels in lung lysates from mice treated with PBS or BLM, with or without RR-11A. β-Actin serves as loading control. (C to F) Quantitative RT-PCR analysis of fibrotic gene expression (*Col1a1* and *Fn1*), hydroxyproline content (a measure of collagen deposition), lung weight, and white blood cell count in bronchoalveolar lavage fluid (WBC in BALF) (*n* = 8 per group; **P < 0.01, ***P < 0.001). (G) Micro-CT imaging and histopathological assessment (H&E and trichrome staining) of lung architecture. Scale bars: 150 μm. (H) Schematic representation of the *Lgmn* flox allele and Cre-mediated recombination strategy. Macrophage-specific *Lgmn* conditional knock-out (KO) mice were generated using *Cx3cr1-CreERT2*; *Lgmn^flox^/^flox^* animals, while controls were *Cx3cr1-CreERT2*; *Lgmn^wt^/^wt^*. (I to L) Assessment of fibrotic markers in macrophage-specific *Lgmn* KO mice. *Col1a1* and *Fn1* mRNA levels, hydroxyproline content, lung weight, and WBC in BALF were significantly reduced in BLM-treated *Lgmn* KO mice compared to controls (*n* = 8 per group; **P < 0.01, ***P < 0.001). (M) Micro-CT and histological analysis (H&E and trichrome) of lungs from control and macrophage-specific *Lgmn* KO mice after BLM challenge. Scale bars: 150 μm.

Previous studies have shown that 8- to 12-week-old mice develop peak fibrosis between day 14 after BLM exposure, with *Lgmn* expression reaching its maximum on day 14 (Fig. S4A). Based on these kinetics and the age of the animals used, RR-11A, a seminal, selective, and irreversible LGMN inhibitor, was administered intraperitoneally from day 7 to 14 following BLM challenge to evaluate its therapeutic efficacy and safety (Fig. 4A). The absence of elevated COL1A1 and FN following RR-11A treatment alone indicates its safety, while its ability to mitigate BLM-induced ECM deposition demonstrates its therapeutic efficacy (Fig. 4B). Consistent reductions in ECM marker transcripts in fibrotic lung homogenates further confirmed the antifibrotic efficacy of RR-11A (Fig. 4C). RR-11A also significantly reduced hydroxyproline content, along with decreased lung weight and white blood cell (WBC) counts, in the BLM-induced fibrosis model (Fig. 4D to F). Micro-computed tomography (micro-CT) revealed strikingly fewer pulmonary opacities in RR-11A-treated mice compared with BLM controls, while histological examination demonstrated reduced cystic airspaces, traction bronchiectasis, inflammatory infiltration, and ECM deposition—collectively indicating that RR-11A effectively mitigates fibrotic lung remodeling (Fig. 4G). To determine whether these protective effects were specifically mediated by macrophage-derived LGMN, we generated *Cx3cr1*-Cre^ERT2^; *Lgmn*^flox/flox^ mice were generated to achieve macrophage-restricted deletion of *Lgmn* (Fig. 4H). Similar to the pharmacological inhibition, macrophage-specific *Lgmn* knockout (*Lgmn*-KO) mice displayed markedly lower hydroxyproline levels, BALF WBC counts, lung weights, and ECM gene expression than littermate controls after BLM exposure (Fig. 4I to L). Consistent with these molecular and biochemical improvements, micro-CT imaging showed attenuated pulmonary opacities, and histological analyses revealed decreased parenchymal destruction, inflammatory cell infiltration, and matrix accumulation (Fig. 4M). These results establish macrophage-derived LGMN as a critical effector driving fibrotic remodeling in the lung.

### LGMN promotes degradation of basement membrane collagen IV

**Fig. 5.**
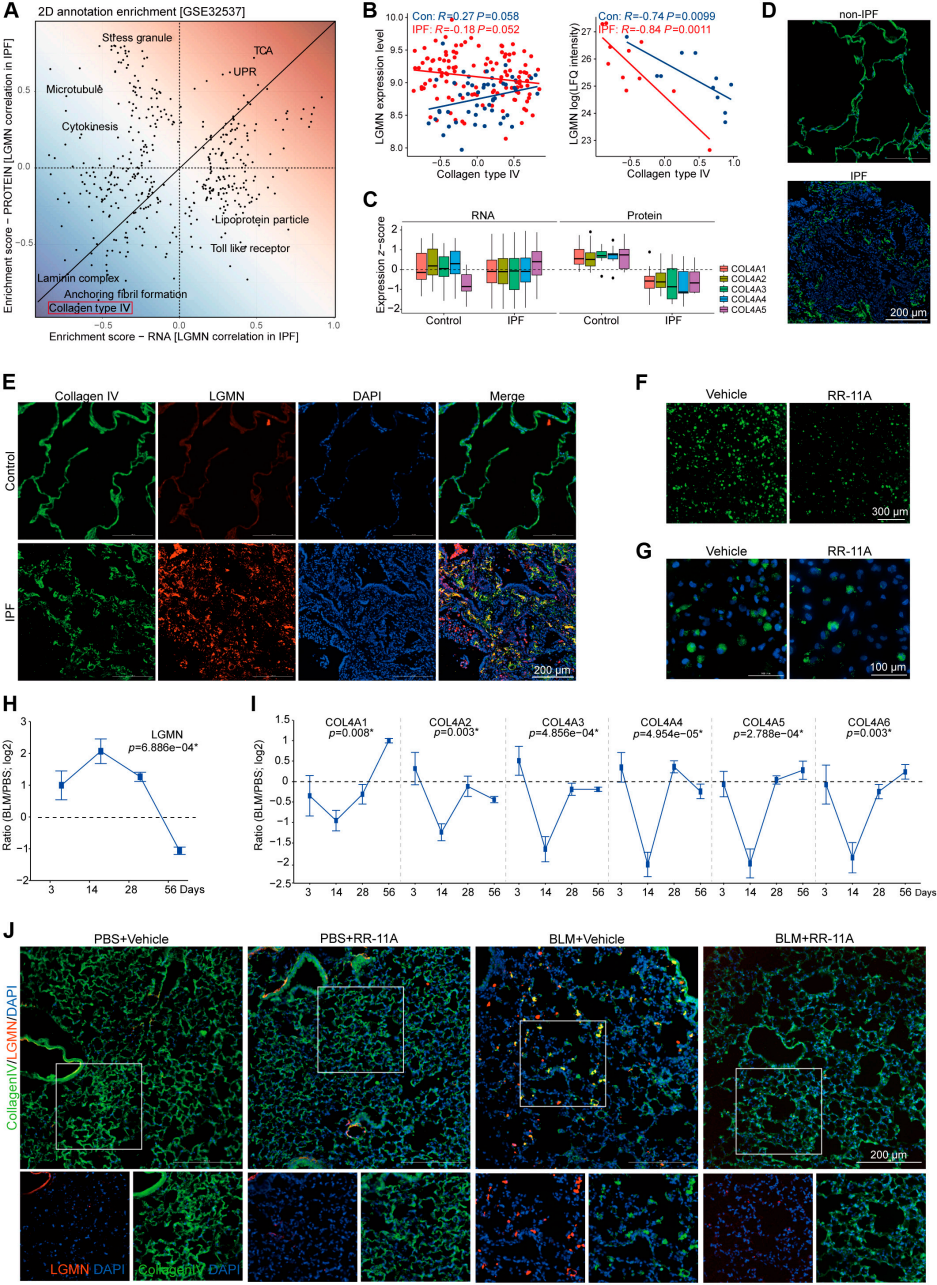
LGMN correlates with extracellular matrix remodeling and collagen deposition in pulmonary fibrosis, and its inhibition attenuates fibrotic progression. (A) Two-dimensional annotation enrichment plot showing gene ontology (GO) terms enriched in genes correlated with LGMN expression in idiopathic pulmonary fibrosis (IPF) patients (GSE32537). Enrichment scores are plotted against RNA-level correlation coefficients. (B) Scatter plots showing correlation between LGMN mRNA expression and collagen type IV levels in lung tissue from control and IPF cohorts. (C) Boxplot comparison of *z*-score normalized expression of COL4A1 to COL4A5 at both RNA and protein levels in control versus IPF lungs. (D) Immunofluorescence staining of COL4A1 (green) in non-IPF and IPF lung tissues. COL4A1 signal is markedly decreased and redistributed in fibrotic areas of IPF samples. Scale bar: 200 μm. (E) Colocalization analysis of collagen IV (green), LGMN (red), and DAPI (blue) in control and IPF lung sections. In IPF, LGMN accumulates in regions of collagen IV deposition, indicating spatial coupling with basement membrane remodeling. Scale bar: 200 μm. (F) Immunofluorescence of DQ-collagen IV in PBMC-derived macrophages after treatment with vehicle or RR-11A. Scale bar: 300 μm. (G) High-magnification view of PBMC-derived macrophages in mouse lung. RR-11A treatment decreases both cell number and DQ-collagen IV fluorescence intensity. Scale bar: 100 μm. (H) Time course of LGMN protein levels relative to PBS control in BLM-treated mice. Expression peaks on day 14 and declines by day 56 (**P < 0.01). (I) Longitudinal quantification of COL4A1 to COL4A6 protein levels in BLM-treated mice (normalized to PBS). All isoforms show dynamic fluctuations, with significant up-regulation at early time points (day 14) and partial resolution by day 56 (*P* < 0.05). (J) Immunofluorescence of collagen IV (green), LGMN (red), and DAPI (blue) in mouse lungs after PBS or BLM challenge with or without RR-11A. In BLM+Vehicle, LGMN colocalizes with collagen IV in fibrotic foci. RR-11A treatment reduces both LGMN accumulation, particularly in alveolar walls. Insets show magnified views of representative regions. Scale bar: 200 μm.

Correlation analysis and 2D pathway enrichment revealed a striking negative association between LGMN and collagen IV at both the mRNA and protein levels (Fig. 5A and Fig. S5A). Gene set variation analysis (GSVA) confirmed that LGMN expression inversely correlated with collagen IV abundance, except in the RNA level of normal tissues (Fig. 5B). Collagen IV subunit transcripts showed minimal changes; however, protein abundance of collagen IV subunits was markedly reduced in human fibrotic lungs (Fig. 5C). Immunostaining demonstrated that collagen IV formed a continuous and intact basement membrane in nonfibrotic tissues, but was severely absent within central fibrotic lesions of IPF patients and fragmented in fibrotic peripheral areas (Fig. 5D). LGMN expression was barely detectable in nonfibrotic tissues, but was markedly up-regulated in fibrotic regions and showed strong spatial colocalization with fragmented collagen IV (Fig. 5E). In vitro, M-CSF-induced PBMC-derived macrophages showed reduced degradation of DQ-collagen IV when treated with the LGMN inhibitor RR-11A, confirming the functional role of LGMN in collagen IV cleavage (Fig. 5F and G).

The murine fibrotic lung exhibited elevated collagen IV mRNA levels alongside reduced protein abundance (Fig. S5B). We next assessed LGMN and collagen IV protein levels in lung tissues from mice with BLM-induced pulmonary fibrosis at multiple time points [37]. LGMN protein levels peaked on day 14 and subsequently declined (Fig. 5H), whereas all 6 collagen IV subunits exhibited the opposite pattern—lowest on day 14 and gradually restored to baseline thereafter (Fig. 5I and Fig. S5C). Immunofluorescence staining of murine lungs revealed continuous collagen IV and minimal LGMN signal in normal lungs, whereas BLM treatment induced fragmented and discontinuous collagen IV, accompanied by robust LGMN expression and pronounced colocalization between LGMN and collagen IV remnants. In contrast, administration of RR-11A to BLM-challenged mice significantly reduced LGMN expression and restored the continuity of the collagen IV network (Fig. 5J). In vitro, RR-11A treatment suppressed the ability of M-CSF-stimulated BMDMs to degrade DQ-collagen IV (Fig. 5J and Fig. S5D). Similarly, in *Lgmn*-KO BMDMs, collagen IV integrity was largely restored in fibrotic lungs, supporting the notion that macrophage-derived LGMN drives collagen IV degradation (Fig. S5E and F). Collectively, these findings demonstrate that *LGMN*^+^ macrophages degrade alveolar–capillary basement membrane collagen IV, thereby disrupting normal respiratory architecture and creating a permissive niche for fibroblast activation and ECM deposition during fibrosis progression.

### CTSS acts as the key effector of LGMN in collagen IV degradation and is a therapeutic target in lung fibrosis

**Fig. 6.**
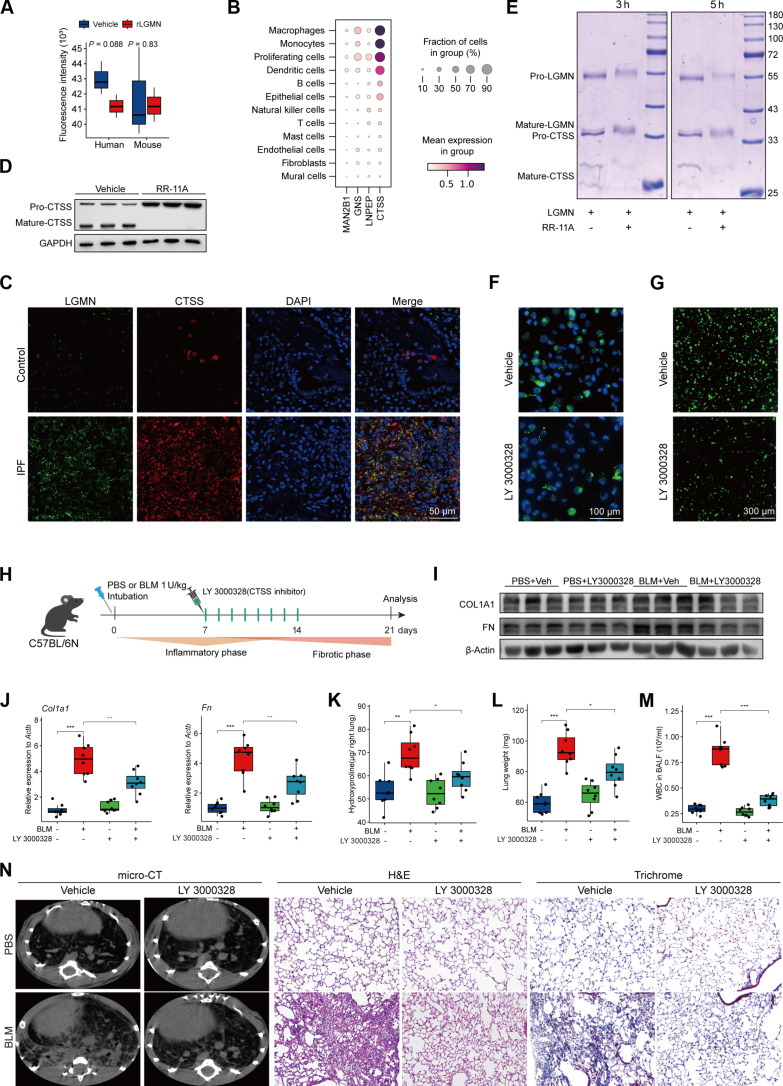
Cathepsin S (CTSS) mediates extracellular matrix remodeling and fibrotic progression in BLM-induced lung injury, independent of LGMN activity. (A) Enzyme-linked immunosorbent assay quantification. No significant change in CTSS processing is observed, indicating that LGMN does not regulate CTSS maturation. (B) Heatmap showing expression profiles of CTSS across cell types in lung tissue from IPF. CTSS is predominantly expressed in macrophages and monocytes. Cell-type proportions and mean expression values are indicated. (C) Immunofluorescence staining of LGMN (green), CTSS (red), and DAPI (blue) in control and BLM-treated mouse lungs. In BLM-treated mice, both LGMN and CTSS colocalize in alveolar macrophages and interstitial infiltrates, suggesting coexpression in profibrotic myeloid populations. Scale bar: 50 μm. (D) Western blot analysis of pro- and mature CTSS protein levels in PBMC-derived macrophages after treatment with vehicle or RR-11A. GAPDH serves as loading control. (E) Coomassie-stained 10% SDS-PAGE gels of recombinant human CTSS incubated with activated recombinant LGMN at pH 5.5 for 3 or 5 h (1:1 CTSS: LGMN mass ratio) in the presence or absence of 100 μM RR-11A. (F and G) Immunofluorescence of DQ-collagen IV in PBMC-derived macrophages treated with vehicle or LY3000328. LY3000328 significantly reduces DQ-collagen IV signal intensity. (H) Experimental timeline showing intratracheal administration of PBS or BLM, followed by daily treatment with LY3000328 or vehicle during the inflammatory and fibrotic phases. Mice were analyzed on day 21 post-BLM. (I) Representative Western blot analysis of COL1A1 and FN protein levels in lung lysates from mice treated with PBS or BLM, with or without LY3000328. β-Actin serves as loading control. (J to M) Quantitative RT-PCR analysis of Col1a1 and Fn1 mRNA expression (J), hydroxyproline content (K), lung weight (L), and white blood cell count in BALF (M) in BLM-treated mice with or without LY3000328. CTSS inhibition significantly reduces fibrotic gene expression, collagen deposition, and inflammation compared to vehicle controls (*n* = 8 per group; **P < 0.01, ***P < 0.001). (N) micro-CT imaging and histopathological assessment (H&E and trichrome staining) of lung architecture. LY3000328 treatment mitigates alveolar wall thickening, collagen accumulation, and structural remodeling induced by BLM. Scale bars: 150 μm.

To determine whether the collagen IV degradation capacity of *LGMN*^+^ macrophages arises from the proteolytic activity of LGMN itself, we incubated recombinant LGMN protein with DQ-collagen IV. Enzyme-linked immunosorbent assay results showed no significant degradation, suggesting that LGMN does not directly cleave collagen IV (Fig. 6A). We therefore explored the mechanism by which LGMN regulates collagen IV degradation. Based on previously published N-Terminomics, we identified 4 potential substrates of LGMN in macrophages [38], among which only *CTSS* is predominantly expressed in macrophages (Fig. 6B). Immunofluorescence staining of human lung tissues revealed that CTSS and LGMN exhibited weak colocalization in relatively normal areas, but strong colocalization in severely fibrotic regions (Fig. 6C). Treatment of PBMC-derived macrophages with the LGMN inhibitor RR-11A markedly reduced the ratio of mature to pro-CTSS protein, as shown by Western blotting (Fig. 6D). In vitro cleavage assays revealed that LGMN activity is required for the generation of this CTSS cleavage product (Fig. 6E). Consistently, inhibition of CTSS significantly diminished the ability of these macrophages to degrade DQ-collagen IV (Fig. 6F and G). scRNA-seq analysis of BLM-treated mouse lungs confirmed that *Ctss* was primarily expressed in macrophages (Fig. S6A). Immunofluorescence analysis further showed that CTSS and LGMN colocalization increased markedly after BLM challenge (Fig. S6B). Proteomic and transcriptomic data demonstrated that CTSS expression peaked on day 14 after BLM exposure—paralleling the increase of LGMN and inversely correlating with collagen IV levels and lung compliance (Fig. S6C to E). In BMDMs, treatment with an LGMN inhibitor reduced CTSS maturation, confirming that LGMN facilitates CTSS activation (Fig. S6G). Moreover, inhibition of CTSS blocked collagen IV degradation in vitro (Fig. S6H and I). We next assessed the therapeutic potential of CTSS inhibition in BLM-induced PF using the selective CTSS inhibitor LY3000328 (Fig. 6H). Based on the temporal expression profile of *Ctss*, LY3000328 was administered intraperitoneally from day 7 to 14 following BLM challenge. Analysis of COL1A1 and FN expression revealed that both transcripts and proteins were markedly elevated after BLM exposure but were restored toward baseline levels upon LY3000328 treatment (Fig. 6I and J). Systemic blockade of CTSS also led to a pronounced reduction in lung hydroxyproline content, accompanied by normalization of lung weight and WBC counts relative to the BLM group (Fig. 6K to M). Micro-CT demonstrated reduced pulmonary opacities in LY3000328-treated mice, while histological staining revealed attenuation of cystic airspaces, traction bronchiectasis, immune-cell infiltration, and ECM accumulation—collectively indicating that CTSS inhibition effectively limits fibrotic remodeling (Fig. 6N). Taken together, these data indicate that LGMN facilitates collagen IV degradation indirectly by activating CTSS and that pharmacological targeting of CTSS can ameliorate PF in vivo.

### LGMN regulates fibroblast proliferation, apoptosis, and activation through integrin α_v_

**Fig. 7.**
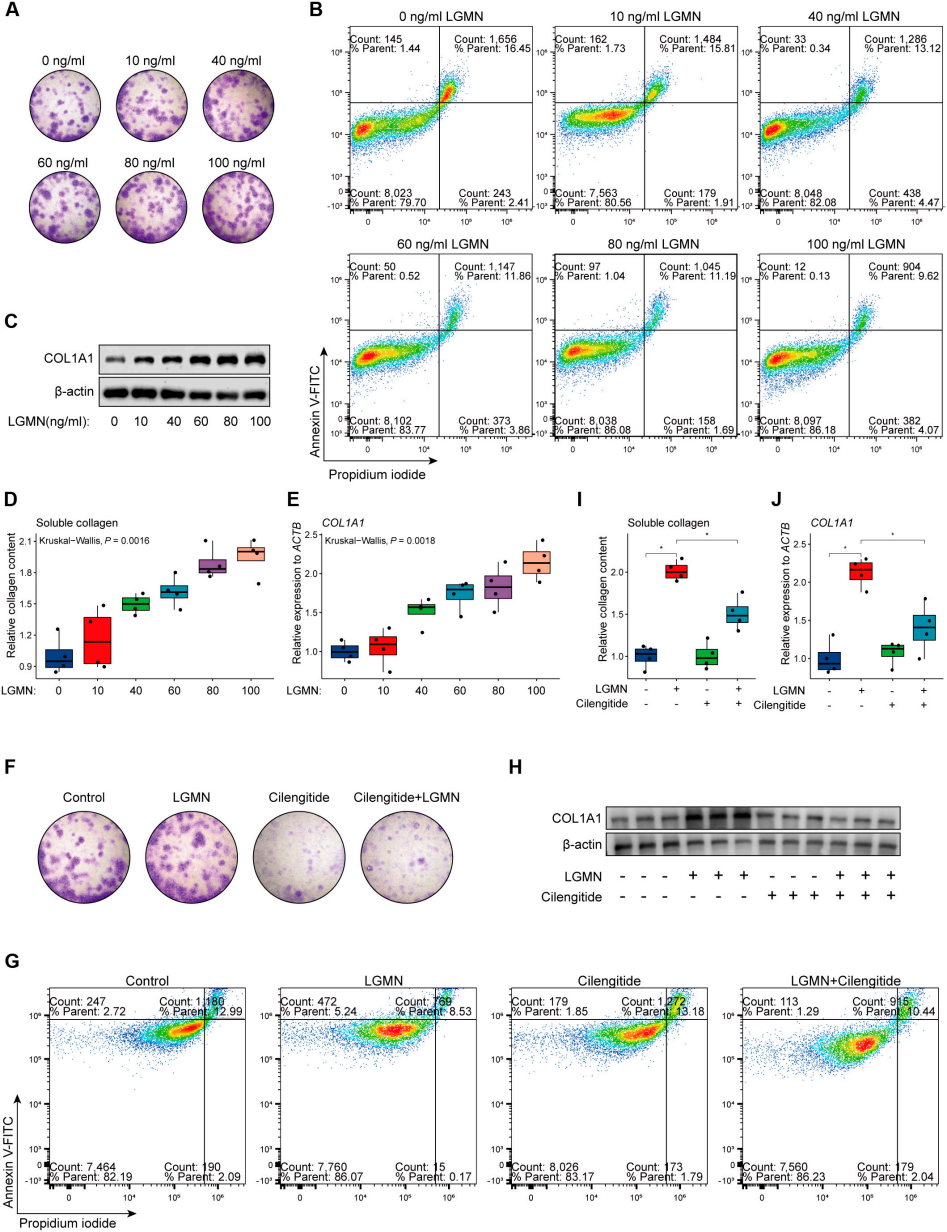
LGMN regulates fibroblast proliferation, apoptosis, and activation through integrin α_v_. (A) Colony formation assay of MRC-5 cells treated with increasing concentrations of recombinant LGMN (0 to 100 ng/ml) for 12 days. (B) Flow cytometric analysis of apoptosis in MRC-5 cells treated with 0, 10, 40, 60, 80, or 100 ng/ml LGMN for 72 h, using Annexin V-FITC/propidium iodide staining. (C) Western blot analysis of COL1A1 and β-actin in MRC-5 cells treated with 0 to 100 ng/ml LGMN for 72 h. (D) Relative COL1A1 mRNA expression (normalized to ACTB) in MRC-5 cells treated with graded LGMN concentrations, as determined by qRT-PCR. Data are presented as box plots (median ± IQR; *n* = 4 independent experiments); statistical significance assessed by Kruskal–Wallis test (*P* = 0.0018). (E) Soluble collagen content in cell culture supernatants of MRC-5 cells treated with LGMN (0 to 100 ng/ml), quantified by Sircol assay. Box plots show median ± IQR (*n* = 4); Kruskal–Wallis test: *P* = 0.0016. (F) Colony formation assay under 4 conditions: untreated control, LGMN, cilengitide, or LGMN+cilengitide. (G) Flow cytometry analysis of apoptosis in MRC-5 cells under the same 4 treatment conditions as in (F). (H) Western blot analysis of COL1A1 and β-actin in MRC-5 cells treated with LGMN, cilengitide, or their combination for 48 h. “+” indicates presence of treatment; “–” indicates absence. (I) Relative COL1A1 mRNA expression in MRC-5 cells treated with LGMN, cilengitide, or both, normalized to ACTB. Box plots (*n* = 4); Kruskal–Wallis test: *P* < 0.0001. (J) Soluble collagen content in supernatants under the same 4 treatment conditions as in (I). Box plots (*n* = 4); Kruskal–Wallis test: *P* < 0.0001.

To evaluate the impact of LGMN on fibroblast proliferation, MRC-5 cells were treated with increasing concentrations of recombinant LGMN protein. Colony formation assays demonstrated that LGMN significantly enhanced both the size and number of colonies formed by MRC-5 cells in a dose-dependent manner (Fig. 7A), indicating a pro-proliferative effect. In contrast to its proliferative role, LGMN conferred resistance to apoptosis in fibroblasts. Flow cytometric analysis using Annexin V/propidium iodide staining revealed that recombinant LGMN dose-dependently reduced the proportion of apoptotic cells (Fig. 7B), with the percentage of early and late apoptotic cells decreasing from 16.45% (0 ng/ml) to 9.62% (100 ng/ml LGMN). Concomitantly, LGMN treatment up-regulated markers of fibroblast activation: quantitative RT-PCR and Western blotting showed significant increases in COL1A1 protein (Fig. 7C and D) and mRNA expression (Fig. 7E) in a concentration-dependent fashion. Given prior evidence suggesting an interaction between LGMN and integrin α_v_ [39,40], we hypothesized that integrin α_v_ is essential for LGMN-mediated regulation in fibroblasts. To test this, cells were cotreated with cilengitide, a selective integrin α_v_ inhibitor. Cilengitide markedly suppressed colony formation (Fig. 7F), abrogated the antiapoptotic effect of LGMN (Fig. 7G), and attenuated LGMN-induced collagen production and COL1A1 expression (Fig. 7H to J). Collectively, these findings demonstrate that LGMN promotes fibroblast proliferation, enhances resistance to apoptosis, and induces activation—effects that are critically dependent on integrin α_v_ signaling, as evidenced by their reversal upon cilengitide inhibition. Thus, the LGMN–integrin α_v_ axis represents a key regulatory pathway governing fibroblast biological behavior.

## Discussion

**Fig. 8.**
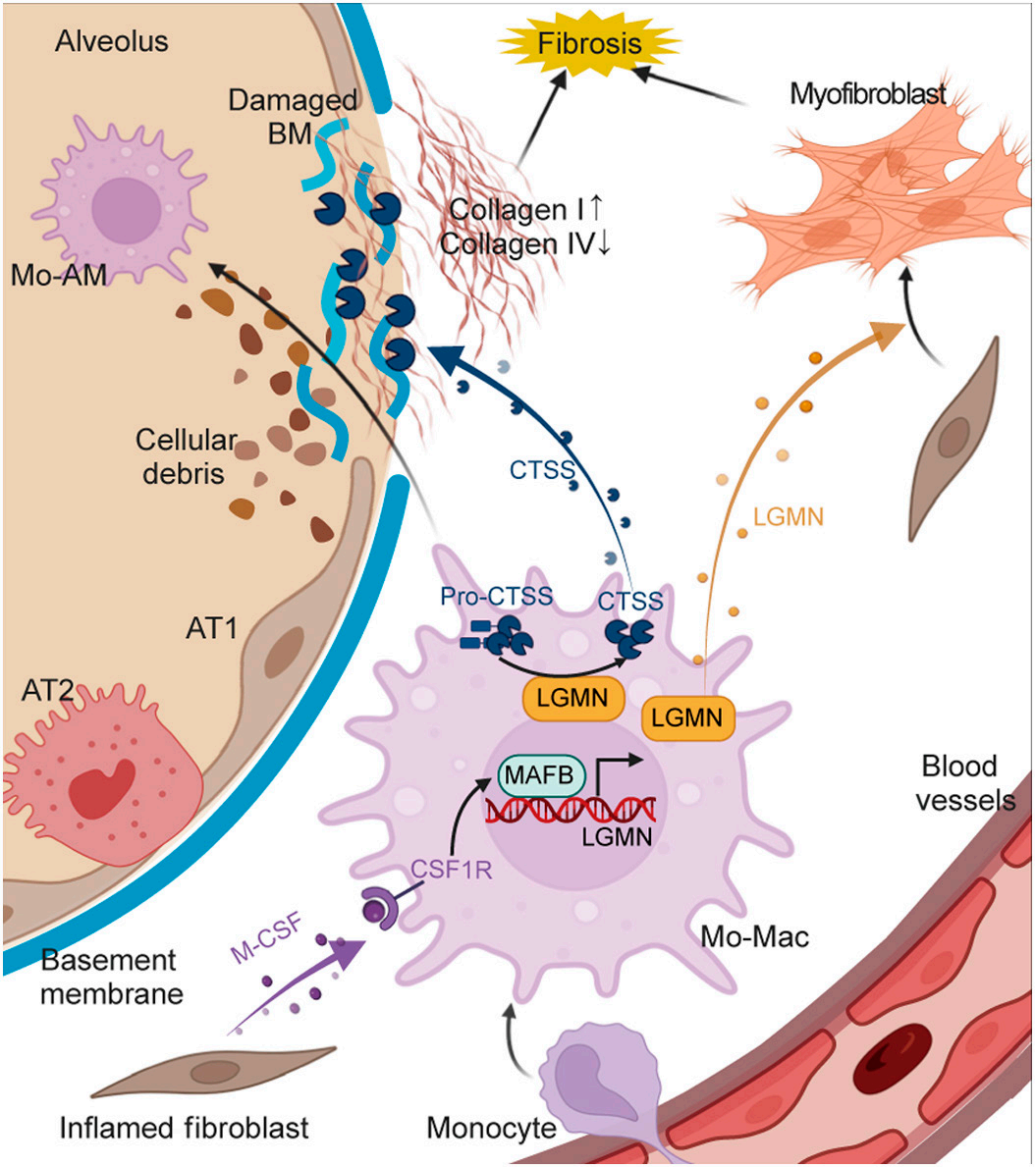
*LGMN*^+^ macrophages drive fibrosis after lung injury. Injured lungs recruit monocytes that differentiate into LGMN-high macrophages via M-CSF and MAFB. These macrophages localize to myofibroblast foci and promote fibrosis by (a) activating CTSS to degrade collagen IV and disrupt alveolar structure, and (b) stimulating fibroblasts to secrete collagen I, replacing normal basement membrane with scar-like matrix. This pathway links immune activation to pathological remodeling in pulmonary fibrosis.

Under homeostatic conditions, resident AMs are self-maintaining and undergo minimal replenishment from circulating monocytes [18]. Upon injury, however, large numbers of monocytes infiltrate the lung and differentiate into monocyte-derived macrophages that shape the outcome of tissue repair [24,25]. Inhibiting this differentiation process, or selectively depleting monocyte-derived macrophages, has been shown to protect against experimental lung fibrosis, highlighting their pathogenic potential [15,25,26,41,42]. In this context, our study identifies a fibrosis-associated macrophage population that is tightly linked to disease progression and poor prognosis. These macrophages, characterized by high expression of *LGMN* and conserved across humans and mice, localize within myofibroblast foci and arise from M-CSF-induced monocyte differentiation driven by MAFB activation. Functionally, LGMN in these macrophages activates CTSS to degrade collagen IV, thereby disrupting the alveolar–capillary structure. Moreover, LGMN directly promotes fibroblast activation and collagen I secretion, facilitating the replacement of collagen IV basement membrane with interstitial collagen I—a key step leading to irreversible architectural distortion and loss of respiratory function in PF. Collectively, these findings delineate a profibrosis macrophage lineage that couples monocyte differentiation to matrix turnover, thereby defining a critical immune checkpoint in the progression from repair to fibrosis [43] (Fig. 8).

Since the recognition of macrophages as central contributors to PF, extensive efforts have been made to precisely define the subsets that drive fibrotic remodeling. Early studies focused on alternatively activated M2 macrophages [44], while later work highlighted monocyte-derived AMs as the dominant profibrotic population [25,42]. With the advent of single-cell transcriptomics, increasingly refined subsets—such as *Cx3cr1^+^SiglecF*^+^ transitional macrophages [26], *CHIT1*^+^ macrophages [28], *SPP1^+^GPNMB*^+^ macrophages [27], and type 3 cytokine-induced “scar-associated macrophages” [29]—were successively proposed. Yet, accumulating evidence has challenged the pathogenic relevance of these subsets, suggesting that many may reflect outcomes of fibrosis rather than drivers of its progression [24,33]. This conceptual discrepancy likely stems from an ambiguous definition of “fibrosis progression”. Most prior studies have compared healthy and diseased tissues and thus fail to capture the dynamic continuum of disease evolution [45]. Clinical indicators such as lung functional decline and patient survival provide a more faithful measure of progression [46,47]. Following this rationale, we identified *LGMN*^+^ macrophages by mapping bulk-seq-derived clinical outcome data onto the single-cell landscape, enabling a progression-based rather than state-based definition of profibrotic macrophages. Notably, LGMN^+^ macrophages have also been independently reported in severe COVID-19 [48], acute respiratory distress syndrome [49], and pulmonary hypertension [50], where their abundance correlates with impaired lung function, further supporting their pathological relevance. Beyond lung injury, *LGMN* expression is associated with adverse prognosis across multiple cancers—including gliomas [51], thymomas, colorectal carcinoma, melanoma, and renal clear cell carcinoma—and promotes fibrogenesis through TGF-β1 activation in chronic pancreatitis [52].

Monocyte-derived macrophages have long been implicated in the initiation and progression of PF [25]. Yet, some studies suggest that infiltrating monocytes may gradually assimilate into the resident macrophage pool to restore pulmonary macrophage homeostasis after injury [25,53]. Our trajectory and lineage-tracing analyses reveal that *LGMN*^+^ macrophages follow an independent differentiation path, distinct from the monocyte-to-resident macrophage transition, thereby representing a unique fate commitment under fibrotic stress. The differentiation of monocytes into macrophages is typically orchestrated by colony-stimulating factors that guide lineage specification. Among them, GM-CSF is essential for maintaining resident AMs [54] and has been implicated in the induction of “scar-associated” macrophages [29]. M-CSF promotes the differentiation of monocyte-derived macrophages and exacerbates fibrosis [42,55]. Consistent with these observations, blockade of M-CSF–CSF1R signaling alleviates BLM-induced PF [42,56]. Our integrative analyses and experimental validation demonstrate that *LGMN*^+^ macrophages originate from M-CSF-activated monocytes. Under normal conditions, M-CSF expression is low and CSF1R on monocytes remains largely masked [57]. Upon tissue injury, IL-1β released by type II alveolar epithelial cells and resident macrophages activates fibroblasts, converting them into inflammatory fibroblasts that secrete abundant M-CSF [58,59]. The resulting activation of CSF1R triggers a transcriptional cascade involving MAF family factors, which are known regulators of terminal differentiation and tissue-repair phenotypes in macrophages [60,61]. Functional perturbation experiments further confirm that MAFB directly promotes *LGMN* transcription in profibrotic macrophages, establishing the M-CSF–MAFB axis as the developmental driver of *LGMN*^+^ macrophage differentiation.

Previous studies have primarily focused on macrophage-derived soluble mediators such as TGF-β that activate fibroblasts and promote collagen deposition [1]. More recently, attention has shifted toward the matrix-remodeling functions of macrophages and their direct contribution to structural alteration [62,63]. In PF, collagen accumulation predominantly involves the so-called “bad collagens”, such as type I and type III, whereas type IV collagen, the principal component of basement membranes, represents the “good collagen” that supports epithelial and endothelial integrity [64,65]. Loss of basement membrane continuity leads to apoptosis of parenchymal cells and impedes the differentiation of progenitors required for gas-exchange unit regeneration [66]. Reduced abundance and disrupted integrity of collagen IV have been consistently observed in both aged mice and patients with fibrosis [37,67,68]. Although neutrophils are known to degrade collagen IV during acute inflammation, this process has not been emphasized in PF dominated by chronic inflammation and mainly composed of macrophages [62,69]. Our data and those of others now identify macrophages as active participants in collagen IV degradation [62,68]. *LGMN* expression inversely correlates with collagen IV levels, and both in vitro and in vivo analyses confirm its role in basement membrane remodeling. Given that *LGMN* requires an acidic lysosomal environment for autocatalytic activation [70], direct extracellular cleavage of collagen IV is unlikely. Instead, LGMN facilitates the maturation of CTSS in human and mice [38], which mediates efficient collagen IV degradation [71,72]. Other studies have confirmed that CTSS can inhibit and alleviate liver fibrosis [73]. In vivo and in vitro experiments have confirmed that CTSS inhibitors can also directly alleviate PF. In addition, fibrosis increases extracellular LGMN abundance [37], and recombinant LGMN directly enhances fibroblast activation and collagen I secretion, suggesting an additional paracrine signaling function [38]. Collectively, monocyte differentiation into profibrotic macrophages initiates a dual program—collagen IV degradation via the LGMN–CTSS axis and fibroblast activation driving collagen I deposition—resulting in the structural replacement of basement membranes and irreversible architectural distortion characteristic of PF.

Although the intratracheal BLM model is the most widely used and best-characterized preclinical system for studying pulmonary fibrosis, it has well-recognized limitations. Specifically, BLM induces an acute, self-limited injury-repair response that does not fully recapitulate the chronic, progressive, and heterogeneous nature of human IPF. Alternative models, such as those based on silica or asbestos exposure or TGF-β overexpression, also fail to faithfully mimic the repetitive epithelial injury and dysregulated repair that are central to IPF pathogenesis [74,75]. Nevertheless, certain key pathological features are consistently shared between the BLM model and human IPF, including basement membrane disruption and fibroblast activation [76]. By focusing on these conserved biological processes, our study aims to identify mechanisms with potential translational relevance, while acknowledging that findings derived from this model must be interpreted within its inherent temporal and mechanistic constraints. Future validation in human IPF tissues or complementary chronic models will be essential to confirm the broader applicability of our observations.

Several questions remain for future investigation. A key issue is to elucidate how *LGMN*^+^ macrophages interact with epithelial and endothelial cells, and whether their differentiation can be reversed once fibrosis is established. Spatial data support LGMN^+^ macrophage–fibroblast crosstalk but lack resolution to confirm interactions with other nearby cells. We therefore focused on this axis, noting that higher-resolution methods are needed to explore broader cellular interactions. Although our findings demonstrate that short-term inhibition of LGMN or CTSS is therapeutically effective, the long-term modulation of these enzymes requires careful evaluation to avoid compromising physiological ECM turnover or host defense. Moreover, LGMN exhibits context-dependent roles across tissues and diseases: its high expression predicts better outcomes in clear cell renal carcinoma, and LGMN deficiency aggravates renal fibrosis, suggesting cell- and tissue-specific functional diversity [77]. Therefore, optimizing the dosing, formulation (e.g., aerosol delivery), and cellular targeting of LGMN inhibitors will be crucial for translating this pathway into safe and effective therapies for lung repair and fibrosis. Given that CTSS is predominantly expressed in macrophages and that CTSS inhibition has shown antifibrotic efficacy in liver fibrosis models, this pathway could represent a safer and more specific therapeutic avenue for lung repair and fibrosis.

In summary, this study delineates a conserved macrophage subset that orchestrates the fibrotic remodeling process through coordinated cellular and molecular programs. At the cellular level, injury-induced AT2s and AMs secrete inflammatory factors that stimulate stable fibroblasts to inflammatory fibroblasts [58,59], which secrete M-CSF to drive the differentiation of monocytes into profibrotic *LGMN*^+^ macrophages. These macrophages, in turn, activate fibroblasts and degrade the “good” basement membrane collagen IV, thereby creating both the signaling cues and structural space necessary for the deposition of “bad” collagens such as type I collagen. At the molecular level, our findings establish the M-CSF–MAFB–LGMN–CTSS axis as a central regulatory pathway that integrates macrophage differentiation, fibroblast activation, and matrix remodeling. Together, this work provides a comprehensive framework linking immune–stromal interactions to the architectural transition from repair to fibrosis, offering mechanistic insight and therapeutic opportunities for halting fibrotic disease progression.

## Methods

In adherence to the ARRIVE guidelines, all animal experiments were performed under protocols authorized by Henan Normal University’s Institutional Animal Care and Use Committee (HTU2023-06).

### Diagnosis and clinical characterization of IPF

IPF was diagnosed according to established international guidelines. For IPF cases derived from public databases, diagnoses were originally made by the contributing institutions in accordance with the ATS/ERS/JRS/ALAT Clinical Practice Guideline [78–80]. In our own cohort, IPF diagnoses were confirmed by experienced interstitial lung disease specialists from Henan Provincial Chest Hospital and Nanjing Drum Tower Hospital. All cases were carefully evaluated to exclude known etiologies such as environmental exposures, connective tissue diseases, or drug toxicity. Diagnosis was primarily based on (high-resolution computed tomography) HRCT findings consistent with a (usual interstitial pneumonia) UIP pattern. Key HRCT features included subpleural and basal-predominant honeycombing, often accompanied by traction bronchiectasis and traction bronchiolectasis. In patients who underwent surgical lung biopsy, histopathological examination further demonstrated the alternating areas of dense scarring and relatively preserved lung parenchyma, along with significant architectural distortion, including destructive fibrosis and/or honeycombing. Comprehensive baseline clinical characteristics, including age, sex, smoking history, pulmonary function (forced vital capacity [FVC] and diffusing capacity of the lung for carbon monoxide [DLCO]), gender–age–physiology (GAP) stage, and treatment exposure, are provided in Table S1.

### Animals

All experiments utilized wild-type C57BL/6N mice (8 weeks of age) sourced from Charles River Laboratories (Beijing, China). Animals were housed under specific pathogen-free conditions, maintained at 22 to 26 °C with 40% to 60% relative humidity and a standard 12-h light/dark cycle. Standard rodent chow (Xietong Organism, Jiangsu; Cat# 1010088) and autoclaved water were provided ad libitum. The *Cx3cr1*-Cre^ERT2^ and *Rosa26R*-CAG-lsl-tdTomato (R26R-tdT) reporter strains have been previously characterized in the literature. Lgmn-floxed (lgmn^f/+^) mice were custom-generated by Cyagen Biosciences (Suzhou, China) and subsequently bred in-house with Cre-expressing lines to generate conditional knockout models, as schematized in Fig. 3G. For lineage-tracing experiments using *Cx3cr1*-Cre^ERT2^; R26R-tdT mice, and for conditional deletion of *Lgmn* in *Cx3cr1*-Cre^ERT2^; lgmn^f/f^ animals, tamoxifen (Sigma-Aldrich; 100 mg/kg body weight, dissolved in Mazola corn oil) was administered via intraperitoneal injection to age-matched cohorts (8 to 12 weeks old), following the dosing schedules indicated in relevant figures. Animals were humanely euthanized at designated time points for tissue collection. Although both male and female mice were included in initial pilot studies—due to known sex-based differences in body mass and BLM sensitivity—biological sex was not treated as an independent experimental variable. Parallel experiments conducted separately in each sex yielded consistent phenotypic outcomes; therefore, all representative data shown are derived from male mice to ensure uniformity in presentation.

### Lung injury models and treatments

Mice were anesthetized using isoflurane delivered via a small-animal anesthesia system (RWD Life Science, Model R550, Shenzhen, China). Following induction, animals were positioned vertically by securing the incisors to an endotracheal intubation platform to facilitate intratracheal instillation. A single dose of BLM (Hanhui Pharmaceutical Co., Zhejiang, China; 1 U/kg body weight) in 50 μl of phosphate-buffered saline (PBS) was administered directly into the trachea through a 22-gauge catheter. For pharmacological interventions, RR-11A (Bide Pharmatech, Shanghai, China) and LY3000328 (MedChemExpress, Cat# HY-15533) were initially solubilized in dimethyl sulfoxide (DMSO) at a stock concentration of 5 mg/ml, then serially diluted in PBS to achieve the target working concentrations. Each mouse received 0.2 ml of the prepared solution via intraperitoneal injection for systemic delivery. Control cohorts were treated identically but received vehicle-only formulations (equivalent volumes of DMSO/PBS mixture) following the same administration schedule.

### Drug dosing rationale

The doses and concentrations of pharmacological inhibitors used in this study were selected based on previously published protocols with demonstrated efficacy and safety in relevant disease models. For in vivo administration, RR-11A (an LGMN inhibitor) was administered at 20 mg/kg, a dose shown to be well-tolerated and effective in murine models of cardiac fibrosis, kidney injury, glioblastoma, and breast cancer [50–52,81–84]. LY3000328 (a CTSS inhibitor) was given at 30 mg/kg, consistent with regimens used in mouse models of pulmonary inflammation and renal ischemia–reperfusion injury [85,86]. For in vitro experiments, BMDMs were treated with RR-11A at 20 nM and LY3000328 at 5 μM concentrations [39,51,85]. Cilengitide, used exclusively in MRC5 fibroblast assays, was applied at 500 nM, a dose widely adopted for targeting αvβ3/αvβ5 integrins in fibroblast and tumor cell contexts [87].

### Measuring the hydroxyproline and BALF cell count

Pulmonary hydroxyproline content was quantified using a colorimetric assay kit (Sigma-Aldrich, Cat# MAK008), strictly following the manufacturer’s protocol. Final values are reported as micrograms of hydroxyproline per whole lung, consistent with data presentation in the corresponding figures. For bronchoalveolar lavage, mouse lungs were sequentially flushed twice with 0.7 ml of sterile saline, and the recovered fluid was pooled for downstream analysis. Total leukocyte counts were determined using an automated hematology analyzer (Beckman Coulter DxH 500).

### Immunoblotting

Total protein was extracted from cultured cells and lung tissue homogenates using radio immunoprecipitation assay (RIPA) lysis buffer (Beyotime Biotechnology, Cat# P0013B). Protein concentrations were determined with a bicinchoninic acid (BCA) assay kit (Solarbio Life Sciences) according to the manufacturer’s protocol. Equal amounts of protein were resolved by sodium dodecyl sulfate–polyacrylamide gel electrophoresis (SDS-PAGE) and subsequently electrotransferred onto polyvinylidene fluoride membranes. Immunoreactive bands were visualized using either a ChemiDoc XRS+ Imaging System (Bio-Rad Laboratories) or an ODYSSEY Fc Imager (LI-COR Biosciences), depending on the detection method employed. The following primary antibodies were applied at a dilution of 1:1,000: Anti-COL1A1 (Cell Signaling Technology, #72026), Anti-FN1 (Proteintech, #15613), Anti-LGMN (Santa Cruz Biotechnology, sc-166971), Anti-MAFB (Proteintech, #20189-1-AP), and Anti-CTSS (Proteintech, #27538-1-AP). Corresponding horseradish peroxidase (HRP)-conjugated secondary antibodies (Affinity Biosciences) were used at 1:5,000 dilutions: Goat anti-Rabbit IgG (H+L) (#S0001) and Goat anti-Mouse IgG (H+L) (#S0002). For fluorescence-based detection, IRDye 800CW-conjugated goat anti-mouse IgG (H+L) (LI-COR, 1:10,000 dilution) was employed.

### Histology and immunostaining

Mouse lungs were systemically cleared of circulating blood cells via transcardiac perfusion through the right ventricle using 10 ml of ice-cold PBS. Immediately thereafter, tissues were subjected to initial fixation by immersion in 4% paraformaldehyde (PFA) for 1 h at room temperature under a constant hydrostatic pressure of 25 cm H₂O—a condition calibrated according to established protocols for murine lung volume standardization. Following this step, samples underwent extended postfixation in fresh PFA at 4 °C overnight, then were exhaustively rinsed in PBS (3 changes, 4 h each, 4 °C) to ensure complete removal of residual fixative. For histological paraffin embedding, lung tissues were progressively dehydrated through an ascending ethanol series, followed by xylene clearing and paraffin infiltration using standard histotechnical procedures. For cryosectioning compatibility, tissues destined for optimal cutting temperature compound (OCT) embedding (Tissue-Tek) were cryoprotected by equilibration in a stepwise sucrose gradient (15% → 30% w/v) at 4 °C prior to snap-freezing. Human lung specimens were obtained from Nanjing Drum Tower Hospital with institutional ethics approval. IPF samples originated from diagnostic surgical biopsies or explanted lungs collected during transplantation. Control tissues were derived from macroscopically and histologically confirmed nondiseased regions adjacent to resected lung tumors. Written informed consent was secured from all participating donors in accordance with institutional and national ethical guidelines.

Following heat-induced antigen retrieval in Tris-EDTA buffer (pH 9.0), tissue sections were incubated for 1 h at room temperature in a blocking solution composed of 5% goat serum, 5% donkey serum, and 0.3% Triton X-100 in PBS to minimize nonspecific binding. Sections were then probed overnight at 4 °C with the following primary antibody: Anti-LGMN (Santa Cruz Biotechnology, sc-166971; dilution 1:100). Signal amplification was achieved using biotin-conjugated goat anti-mouse IgG (H+L), followed by incubation with HRP-conjugated streptavidin (Beyotime, Cat# A0288). Antigen–antibody complexes were visualized using 3,3′-diaminobenzidine chromogen. Nuclei were counterstained with hematoxylin, and slides were dehydrated, cleared, and mounted for brightfield microscopy.

Cryosections or paraffin sections (8 to 10 μm thickness) were subjected to standard washing and blocking procedures prior to primary antibody incubation. The following primary antibodies were applied at the indicated dilutions: Anti-CD68 (Proteintech, #84596-4-RR; 1:200), Anti-F4/80 (Proteintech, #28463-1-AP; 1:200), Anti-LGMN (Santa Cruz Biotechnology, sc-166971; 1:100), Anti-COL4A1 (Abcam, #ab6586; 1:200), and Anti-CTSS (Proteintech, #27538-1-AP; 1:100). After thorough washing, sections were incubated for 60 min at room temperature with species-matched fluorescent secondary antibodies: Alexa Fluor 488-conjugated anti-rabbit IgG (Cell Signaling Technology, #4416; 1:1,000) and Alexa Fluor 594-conjugated anti-mouse IgG (Cell Signaling Technology, #8889; 1:1,000). Nuclear counterstaining was performed using 4′,6-diamidino-2-phenylindole (DAPI). Slides were coverslipped with antifade mounting medium (Beyotime, Cat# P0126) to preserve fluorescence signal integrity. Fluorescent images were acquired using a Leica TCS SP8 confocal laser scanning microscope equipped with an oil-immersion objective (numerical aperture 1.4). For collagen IV-stained samples, low-magnification overviews were captured using a BioTek Cytation C10 automated imaging system to ensure comprehensive architectural context across entire tissue sections.

### Quantitative RT-PCR

Total RNA was isolated from tissues or cultured cells using TRIzol reagent (Invitrogen, Thermo Fisher Scientific) following the manufacturer’s protocol. RNA integrity and concentration were assessed spectrophotometrically prior to reverse transcription. Complementary DNA (cDNA) was synthesized from equal amounts of total RNA using the PrimeScript RT Reagent Kit (Takara Bio, Cat# RR036A), which includes gDNA removal and first-strand synthesis steps. qPCR was performed with SYBR Green Master Mix (Yesen Bioscience, Cat# 11201) on a Roche LightCycler 480 II Real-Time PCR System under standard cycling conditions. The primer sequences used are listed below:

Human targets:

LGMN

F: 5′-CCTGAAGATGGAGGCAAGCACT-3′

R: 5′-GTTCGTCAGGAATCCCATTGCG-3′

COL1A1

F: 5′-GATTCCCTGGACCTAAAGGTGC-3′

R: 5′-AGCCTCTCCATCTTTGCCAGCA-3′

FN1

F: 5′-ACAACACCGAGGTGACTGAGAC-3′

R: 5′-GGACACAACGATGCTTCCTGAG-3′

Mouse targets:

Lgmn

F: 5′-ATCAACCGACCTAACGGCACAG-3′

R: 5′-ACAGCTTCTGCGTCACCTCTCA-3′

Col1a1

F: 5′-CCTCAGGGTATTGCTGGACAAC-3′

R: 5′-CAGAAGGACCTTGTTTGCCAGG-3′

Fn1

F: 5′-CCCTATCTCTGATACCGTTGTCC-3′

R: 5′-TGCCGCAACTACTGTGATTCGG-3′

### Micro-CT scanning

In vivo micro-CT imaging was performed using a Skyscan 1276 system (Bruker, Belgium) under isoflurane anesthesia to ensure physiological stability during scanning. Mice were positioned in the supine position, and data acquisition was conducted with the following settings: x-ray source filter: 0.5 mm aluminum (Al), voxel resolution: 13 μm, rotation step: 0.4° per projection, frame averaging: 2 frames per angle. Raw projection images were reconstructed into cross-sectional slices using NRecon software (Bruker), applying beam-hardening correction and consistent smoothing parameters across all samples to ensure comparable image quality.

### Flow cytometry

Lung tissues from Cx3cr1-CreERT2; R26R-tdT mice were dissociated using the protocol established for monocyte/macrophage isolation. Following digestion, red blood cells were lysed with an ammonium–chloride–potassium lysis buffer (Beyotime, Cat# C702). Cells were then centrifuged at 400 × *g* for 5 min, washed, and fixed in 4% PFA for 10 min at room temperature. After fixation, samples were permeabilized with 0.5% Tween 20 in PBS for 10 min under the same conditions. For intracellular staining, cells were blocked and incubated with primary antibody against LGMN (Santa Cruz Biotechnology, sc-166971; dilution 1:100) in PBS containing 0.1% Tween 20 and 3% bovine serum albumin for 1 h. Subsequently, Alexa Fluor 488-conjugated secondary antibody (Invitrogen, A-11008) was applied and incubated in the dark for 45 min to detect bound primary antibody. Labeled cells were analyzed and sorted using a BD FACSVerse flow cytometer. tdTomato-positive (tdT+) lineage-labeled cells were selected as the population of interest, within which the proportion of LGMN+ cells was quantified.

### Isolation and differentiation of human monocytes

PBMCs were isolated from fresh whole blood using density gradient centrifugation. Briefly, blood was diluted 1:1 with PBS (pH 7.4) and carefully layered over Ficoll-Paque PLUS (GE Healthcare) in a 15-ml conical tube. Samples were centrifuged at 400 × *g* for 30 min at room temperature with the brake disengaged to preserve interface integrity. The PBMC-rich layer at the plasma–Ficoll interface was collected, washed twice with PBS (300 × *g*, 10 min per wash), and resuspended in RPMI-1640 medium. Monocyte enrichment was achieved through adherence-based selection. Cells were plated and incubated for 1 to 2 h at 37 °C under 5% CO₂; nonadherent cells (primarily lymphocytes) were removed by gentle washing with prewarmed PBS. Adherent primary monocytes were then seeded at a density of 3 × 10^5^ cells per well in flat-bottom, tissue culture-treated 96-well plates (BD Falcon). Cultures were maintained in RPMI-1640 supplemented with 10% fetal bovine serum (FBS), 10 mM HEPES, and 2 mM GlutaMAX (all Gibco). To induce differentiation into macrophages, recombinant human GM-CSF or M-CSF (MedChemExpress; 10 ng/ml) was added immediately after plating. Cytokine-supplemented media were refreshed on day 3, and cells were cultured for a total of 7 days under normoxic conditions (37 °C, 5% CO₂).

### Isolation and culture of murine bone marrow-derived progenitor cells

Female C57BL/6 mice were euthanized via CO₂ asphyxiation followed by cervical dislocation. To minimize microbial contamination, carcasses were briefly immersed in 70% ethanol prior to dissection. Skin and surrounding muscle tissues were meticulously excised to ensure high purity of bone marrow harvest. Femurs and tibias were aseptically dissected by severing the Achilles tendon, fibula, and knee joint. Bones were briefly dipped in 70% ethanol for surface sterilization, then rinsed with ice-cold PBS (5 min, 4 °C). Epiphyses were removed using fine ophthalmic scissors to expose the marrow cavity. Bone marrow was flushed from individual femurs and tibias into a 50-ml conical tube using approximately 5 ml of cold RPMI-1640 per bone, supplemented with 2% FBS and 1% antibiotic–antimycotic solution. Flushing was performed slowly with a 10-ml syringe fitted with a 23-gauge needle to avoid cell lysis. Collected marrow suspensions were pooled, filtered through a 100-μm cell strainer to remove debris, and centrifuged (300 × *g*, 5 min, 4 °C). The resulting pellets were resuspended in complete RPMI-1640 medium containing 10% FBS, 1% antibiotic–antimycotic, and either 20 ng/ml M-CSF or GM-CSF (MedChemExpress). Cells were seeded into culture plates and differentiated for 5 consecutive days under standard conditions (37 °C, 5% CO₂).

### siRNA transfection

Cells were transfected using Lipofectamine 3000 (Invitrogen, Cat# L3000015) following the manufacturer’s protocol with minor adaptations. Prior to transfection, cells were seeded into 6-well tissue culture plates and grown to approximately 70% to 80% confluence in complete growth medium without antibiotics. For each well, siMAFB was first diluted in 125 μl of Opti-MEM Reduced-Serum Medium (Gibco), then combined with 5 μl of P3000 Enhancer Reagent. In a separate tube, 5 μl of Lipofectamine 3000 reagent was diluted in an equal volume of Opti-MEM. After incubation at room temperature for 10 to 15 min, the nucleic acid–lipid complex mixture was added dropwise to the cells while gently swirling the plate to ensure uniform distribution. Six hours posttransfection, the transfection medium was replaced with fresh complete growth medium to minimize cytotoxicity. Cells were subsequently harvested 24 to 48 h after transfection for downstream analyses, depending on the experimental endpoint.

### Batch effect assessment and correction of public single-cell datasets

This study integrated multiple IPF single-cell RNA-seq datasets from public repositories, totaling 301,163 cells from 6 studies: Kaminski_2020 (*n* = 144,404), Banovich_Kropski_2020 (*n* = 57,668), Sheppard_2020 (*n* = 33,817), Lafyatis_2019 (*n* = 32,241), Schiller_2020 (*n* = 17,525), and Misharin_Budinger_2018 (*n* = 15,508). All samples were clinically confirmed as IPF. Metadata included dataset source, sample ID, donor ID, sequencing platform, processing site, and demographic characteristics. Batch effects were assessed using Scanpy. Data were normalized by library size scaling to the median total count. Principal component analysis (PCA) was performed on the normalized matrix to compute the first 50 PCs. Potential batch covariates (dataset, sample, donor_id, cell type, platform, processing site, sequencing direction, age, sex, mitochondrial fraction, ribosomal fraction, and total counts) were evaluated. Technical covariates (platform and processing site) were shuffled 10 times to generate negative controls. Linear regression models quantified the variance explained by each covariate across the 50 PCs. Total explained variance was normalized and ranked to identify dominant batch effects. Shuffled covariates’ mean explained variance and standard deviation were calculated as random effect benchmarks. Integration algorithms were benchmarked using the SCIB framework [35]. Multiple metrics balanced batch removal and biological variance conservation: Batch removal: kBET (batch mixing), graph iLISI (local batch diversity), ASW (batch silhouette), graph connectivity (cell type continuity), and PCR (batch variance in PCs). Biological conservation: Label conservation (NMI, ARI, cell-type ASW, and isolated label scores) and label-free conservation (cell-cycle variance, HVG overlap, and trajectory correlation). A weighted average score ranked algorithms. Integration was performed in Python using scib, with parameter settings following best practices. Downstream analyses used corrected expression matrices or low-dimensional embeddings as appropriate.

### scRNA-seq data preprocessing and analysis

Raw gene expression matrices were processed using Scanpy (v1.9.1), a Python-based toolkit for single-cell genomics. Initial quality control excluded cells expressing fewer than 500 genes or exhibiting more than 20% mitochondrial transcript content, indicative of low viability or cellular stress. Genes detected in fewer than 300 cells were removed to retain only robustly expressed transcripts. To correct for technical variation arising from differences in library size and sequencing depth, count data were normalized using the SCRAN algorithm, which employs pooling-based size factor estimation suitable for heterogeneous cell populations. For downstream analysis, dimensionality reduction was performed via PCA on highly variable genes, followed by uniform manifold approximation and projection (UMAP) embedding. Cell clustering was conducted using the Louvain algorithm applied to a shared nearest neighbor graph constructed at a resolution optimized for biological coherence. Differential gene expression analysis was carried out within Scanpy. For each cell type, average gene expression levels—aggregated per patient and log-transformed—were compared both pairwise against all other clusters and collectively against the remaining population. Marker genes were defined as those showing a minimum fold change of 3 and statistically significant differential expression (adjusted *P* value < 0.05). Only genes meeting these criteria in intercluster comparisons were retained as putative identity markers. Trajectory inference was performed using Slingshot to reconstruct developmental or activation pseudotime paths. RNA velocity analysis was conducted with Velocyto.py to infer dynamic changes in spliced versus unspliced mRNA ratios, providing insights into transcriptional directionality. Regulatory network inference was implemented through pySCENIC, which identifies coexpression modules and infers TF regulon activity based on motif enrichment and expression correlation, followed by AUCell scoring to determine regulon activity per cell.

### Public bulk mRNA data, prognostic signature, and survival analysis

Bulk transcriptomic datasets were systematically retrieved from the NCBI Gene Expression Omnibus (GEO) database to facilitate a robust cross-cohort analysis. To ensure analytical comparability across these heterogeneous datasets, our preprocessing pipeline was designed to retain only protein-coding genes that were consistently present in all cohorts, thereby establishing a common genomic foundation for downstream integrative analyses. Prior to merging the datasets into a unified expression matrix, each individual cohort underwent gene-wise mean centering. This critical normalization step effectively mitigates technical variability arising from interstudy batch effects and platform-specific biases, allowing for a more accurate assessment of biological signals.

To identify cell type-associated survival-related genes, we implemented a sophisticated cell type-specific prognostic signature analysis framework. This approach capitalizes on gene-pair log ratios as stable, normalization-invariant features. By focusing on the relative expression of one gene to another within the same sample, this method inherently controls for global shifts in expression levels and technical noise, providing a robust measure of transcriptional state that is highly reproducible across different experimental platforms.

The analytical workflow proceeded as follows: candidate prognostic gene pairs were identified using a strategy adapted from Paquet and Hallett [88]. In a key methodological refinement, rather than relying on permutation-based scoring, we employed the statistical significance (*P* values) derived from univariate Cox proportional hazards regression as the primary selection metric. This ensures that the selected gene pairs have a direct and statistically grounded association with patient survival outcomes. The log-transformed ratios of these significant gene pairs were then integrated as features into a multivariable survival model.

For model construction, we utilized GLMNET to implement a regularized Cox regression with an elastic net penalty (*α* = 0.1) [89]. The choice of a low *α* value was deliberate, as it favors a more inclusive model by balancing L1 (lasso) and L2 (ridge) regularization. This hybrid penalty structure is particularly advantageous in high-dimensional genomic data, as it not only performs feature selection but also retains groups of correlated yet biologically informative features that might be jointly predictive of survival.

To guarantee the model’s reliability and generalizability, we adopted a rigorous double cross-validation framework for training and evaluation. An inner loop performed nested cross-validation to optimize the regularization parameter (*λ*), ensuring the model complexity was appropriately tuned to the data. Concurrently, an outer loop was used to assess the final model’s predictive performance and generate unbiased estimates of its error. Critically, all reported results—including the final signature coefficients and their associated survival hazard ratios—are based exclusively on the outcomes of this outer cross-validation cycle. This stringent approach effectively minimizes the risk of overfitting and provides a strong assurance of the signature’s validity and potential for translation to independent clinical cohorts.

### Statistical analysis

Data are summarized using boxplots, with center lines representing the median, bounds indicating interquartile range (IQR), and whiskers extending to 1.5× IQR. Statistical significance was defined as *P* < 0.05. Blinding was not applied during experimental interventions; however, data collection and analysis of mouse-derived samples were performed under blinded conditions to reduce assessment bias. No data points were excluded from any analysis, ensuring full transparency and inclusivity of results. For in vivo efficacy studies, a minimum of 8 mice per group was used, and all animal experiments were independently repeated at least twice to confirm reproducibility. In vitro assays were conducted across a minimum of 3 independent biological replicates unless otherwise indicated. Group comparisons between 2 conditions were evaluated using either a 2-tailed unpaired Student’s *t* test (for normally distributed data) or the Mann–Whitney *U* test (for nonparametric data), depending on distributional assumptions. Survival outcomes were assessed using Kaplan–Meier estimates and compared by the log-rank (Mantel–Cox) test. All statistical analyses were carried out in R (v4.4).

## Data Availability

Human scRNA-seq datasets were obtained from the Gene Expression Omnibus (GEO) under accession numbers GSE135893, GSE136831, GSE122960, GSE128033, and GSE132771. Mouse single-cell datasets from different time points following BLM treatment were derived from Santa et al. RNA splicing datasets provided by Guo et al. (CRA011039) were used for RNA velocity analysis. Human bulk transcriptomic datasets were retrieved from GEO (GSE32537, GSE47460, GSE124685, and GSE150910), and clinical transcriptomic data with survival information were obtained from GSE70867 and GSE70866. Mouse bulk RNA-seq datasets from different time points after BLM challenge were obtained from GSE195773 and GSE218997, and proteomic data were collected from Schiller et al. and PXD041050. Transcriptomic data of M-CSF- and GM-CSF-induced macrophages were obtained from GSE248927, and data of M-CSF-induced macrophages with siMAF or siMAFB treatment were from GSE155719. ChIP-seq data for human MAF and MAFB were obtained from Cistrome. N-Terminomics of LGMN were obtained from Ziegler et al.
