# Supplementary material for: Monocyte-Derived LGMN+ Macrophages Divert Lung Injury Outcomes toward Fibrosis through Matrix Remodeling
Source: Research (Wash D C). 2026 Jun 29;9:1341. doi: 10.34133/research.1341 (PMC13311260; doi:10.34133/research.1341)
Supplement: Supplementary 1 — Figs. S1 to S6 Tables S1 to S5 [file research.1341.f1.zip › Supplementary File1.docx]

**Table 1** Baseline clinical characteristics of IPF patients (GSE32537, n = 119)

| **Parameter** | **No. of patients (n = 119)** |
| --- | --- |
| **Age** |  |
| ≤60 | 44 (37.0%) |
| >60 | 75 (63.0%) |
| **Gender** |  |
| Male | 77 (64.7%) |
| Female | 42 (35.3%) |
| **Smoking Status** |  |
| Nonsmoker | 41 (34.5%) |
| Former smoker | 70 (58.8%) |
| NA | 8 (6.7%) |
| **Pack Years** |  |
| 0 | 41 (34.5%) |
| 1–20 | 23 (19.3%) |
| >20 | 47 (39.5%) |
| NA | 8 (6.7%) |
| **DLCO % predicted** |  |
| ≤60% | 77 (64.7%) |
| >60% | 22 (18.5%) |
| NA | 20 (16.8%) |
| **FVC % predicted** |  |
| ≤80% | 99 (83.2%) |
| >80% | 18 (15.1%) |
| NA | 2 (1.7%) |
| **St. George’s Total Score** |  |
| 0 | 0 (0.0%) |
| 1–50 | 61 (53.8%) |
| >50 | 50 (42.0%) |
| NA | 8 (6.7%) |

**Table 2** Baseline clinical characteristics of IPF patients (GSE47460, n = 160)

| **Parameter** | **No. of patients (n = 160)** |
| --- | --- |
| **Age** |  |
| ≤60 | 47 (29.3%) |
| >60 | 113 (70.6%) |
| **Gender** |  |
| Male | 110 (68.8%) |
| Female | 50 (31.3%) |
| **Smoking Status** |  |
| Current | 2 (1.3%) |
| Ever | 96 (60.0%) |
| Never | 58 (36.3%) |
| NA | 4 (2.5%) |
| **DLCO % predicted** |  |
| ≤60% | 109 (68.1%) |
| >60% | 36 (22.5%) |
| NA | 15 (9.4%) |
| **FVC % predicted (Pre-bd^1^)** |  |
| ≤80% | 130 (81.3%) |
| >80% | 27 (16.9%) |
| NA | 3 (1.9%) |
| **FVC % predicted (Post-bd^2^)** |  |
| ≤80% | 43 (26.9%) |
| >80% | 14 (8.9%) |
| NA | 103 (57.2%) |
| **FEV1 % predicted (Pre-bd)** |  |
| ≤80% | 110 (68.8%) |
| >80% | 47 (29.4%) |
| NA | 3 (1.9%) |
| **FEV1 % predicted (Post-bd)** |  |
| ≤80% | 32 (20.0%) |
| >80% | 25 (15.6%) |
| NA | 103 (64.4%) |
| **Emphysema % (F-950^3^)** |  |
| ≤5% | 73 (45.6%) |
| >5% | 6 (3.8%) |
| NA | 81 (50.6%) |
| **GOLD stage** |  |
| 0 (At Risk) | 55 (34.4%) |
| 1 (Mild) | 0 (0.0%) |
| 2 (Moderate) | 1 (0.6%) |
| 3 (Severe) | 2 (1.2%) |
| 4 (Very Severe) | 0 (0.0%) |
| NA | 102 (63.8%) |
| ^1^Pre-bd means lung function before bronchodilator use, ^2^post-bd means after, and ^3^F-950 is the percentage of lung with emphysema on a full-inhalation CT scan using a −950 HU threshold. | |

**Table 3** Baseline clinical characteristics of IPF patients (GSE124685, n = 10)

| **Parameter** | **No. of patients (n = 10)^1^** |
| --- | --- |
| **Age** |  |
| ≤60 | 7 (70.0%) |
| >60 | 3 (30.0%) |
| **Gender** |  |
| Male | 10 (100.0%) |
| Female | 0 (0.0%) |
| **Pack Years** |  |
| 0 | 0 (0.0%) |
| 1–20 | 5 (50.0%) |
| >20 | 5 (50.0%) |
| **DLCO % predicted** |  |
| ≤60% | 10 (100.0%) |
| >60% | 0 (0.0%) |
| **FVC % predicted** |  |
| ≤80% | 9 (90.0%) |
| >80% | 1 (10.0%) |
| **FEV1 % predicted** |  |
| ≤80% | 9 (90.0%) |
| >80% | 1 (10.0%) |
| ^1^Each patient was sampled six times from regions with different degrees of fibrosis and subjected to sequencing. | |

**Table 4** Baseline clinical characteristics of IPF patients (GSE150910, n = 103)

| **Parameter** | **No. of patients (n = 103)** |
| --- | --- |
| **Age** |  |
| ≤60 | 46 (44.7%) |
| >60 | 57 (55.3%) |
| **Gender** |  |
| Male | 57 (55.3%) |
| Female | 46 (44.7%) |
| **Ever smoked** |  |
| Yes | 55 (53.4%) |
| No | 40 (38.8%) |
| NA | 8 (7.8%) |
| **Race** |  |
| Hispanic | 7 (6.8%) |
| Black | 4 (3.9%) |
| Asian | 2 (1.9%) |
| White | 85 (82.5%) |
| Other | 3 (2.9%) |
| Unknown | 2 (1.9%) |
| **Genotype rs35705950** |  |
| GG | 54 (52.4%) |
| GT | 43 (41.7%) |
| TT | 6 (5.8%) |
| **Sample Type** |  |
| Biopsy | 36 (35.0%) |
| Explant | 67 (65.0%) |

**Table 5** Baseline clinical characteristics of IPF patients (GSE70867, n = 176)

| **Parameter** | **No. of patients (n = 176)** |
| --- | --- |
| **Age** |  |
| ≤60 | 35 (19.9%) |
| >60 | 141 (80.1%) |
| **Gender** |  |
| Male | 144 (81.8%) |
| Female | 32 (18.2%) |
| **Cohort** |  |
| Freiburg | 62 (35.2%) |
| LEUVEN | 64 (36.4%) |
| SIENA | 50 (28.4%) |
| **Survival** |  |
| Live | 76 (43.2%) |
| Dead | 100 (56.8%) |
| **Survival Time (Day)** |  |
| < 365 | 51 (29.0%) |
| 365-730 | 60 (34.1%) |
| 731-1095 | 31 (17.6%) |
| >1095 | 34 (19.3%) |
