# Supplementary material for: Monocyte-Derived LGMN+ Macrophages Divert Lung Injury Outcomes toward Fibrosis through Matrix Remodeling
Source: Research (Wash D C). 2026 Jun 29;9:1341. doi: 10.34133/research.1341 (PMC13311260; doi:10.34133/research.1341)
Supplement: Supplementary 1 — Figs. S1 to S6 Tables S1 to S5 [file research.1341.f1.zip › Supplementary Materials.docx]

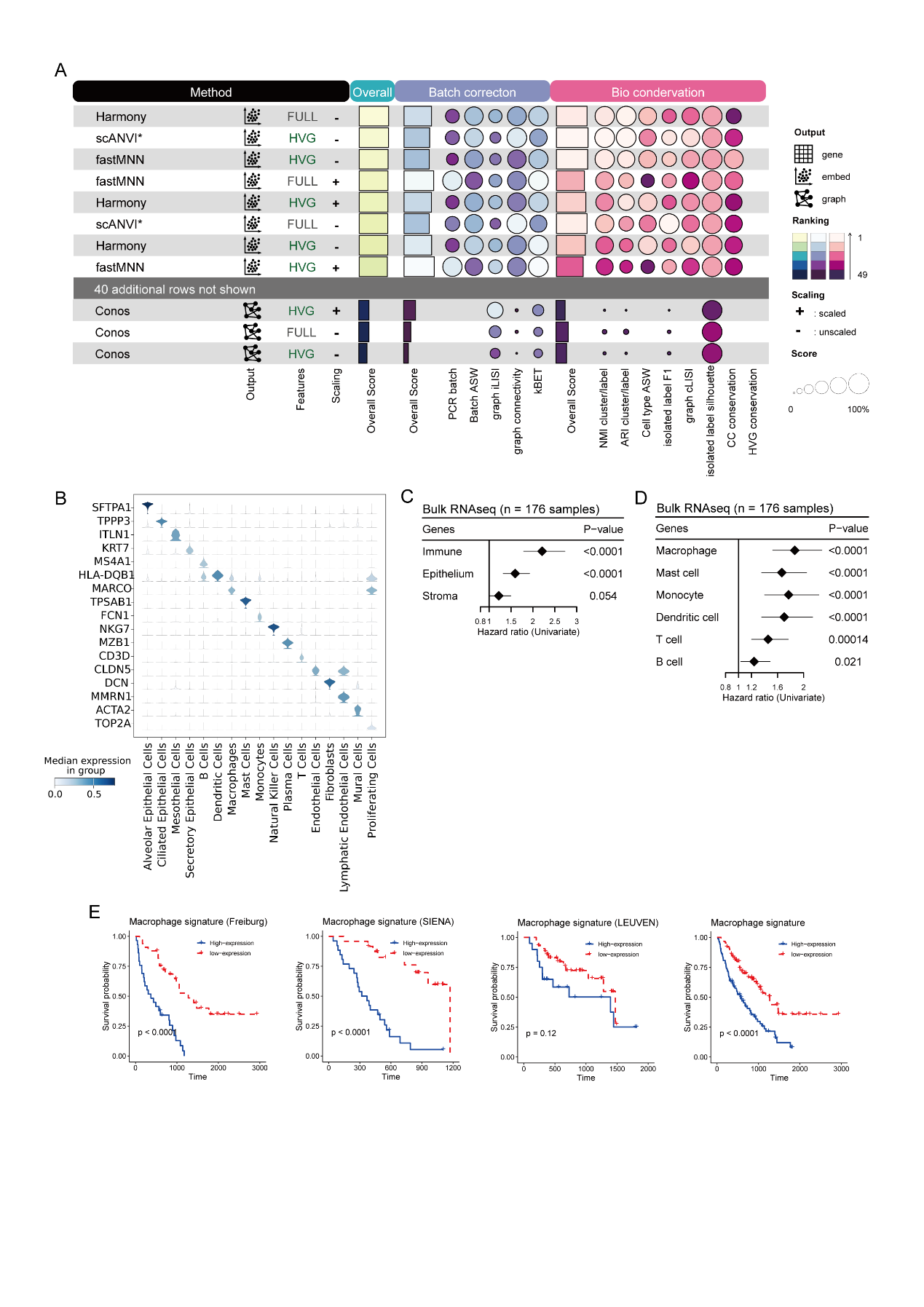


Fig. S1.

(A) Overview of top and bottom ranked methods by overall score for the human lung atlas. Metrics are divided into batch correction (blue) and bio-conservation (pink) categories. Overall scores are computed using a 40/60 weighted mean of these category scores (see Methods for further visualization details and Supplementary Fig. 2 for the full plot). (B) Median expression of selected genes across lung cell types. Dot plot showing gene expression levels (normalized) in various cell populations, with color intensity reflecting median expression. (C) Comparison of three prognostic signatures, each derived using epithelial, stromal, and immune genes only. Univariable Cox regression was used to obtain the hazard ratios (with Wald 95% confidence intervals shown as horizontal bars, and P values given on the right) based on the cross-validated prognostic scores derived using the GLMNET Cox model and applied to pairwise differences of expression of the genes. (D) Similar analysis as in (C), but using genes dominant in each subdivision of the immune cell types. (E) Kaplan-Meier plot of cross-validated macrophage prognostic score.


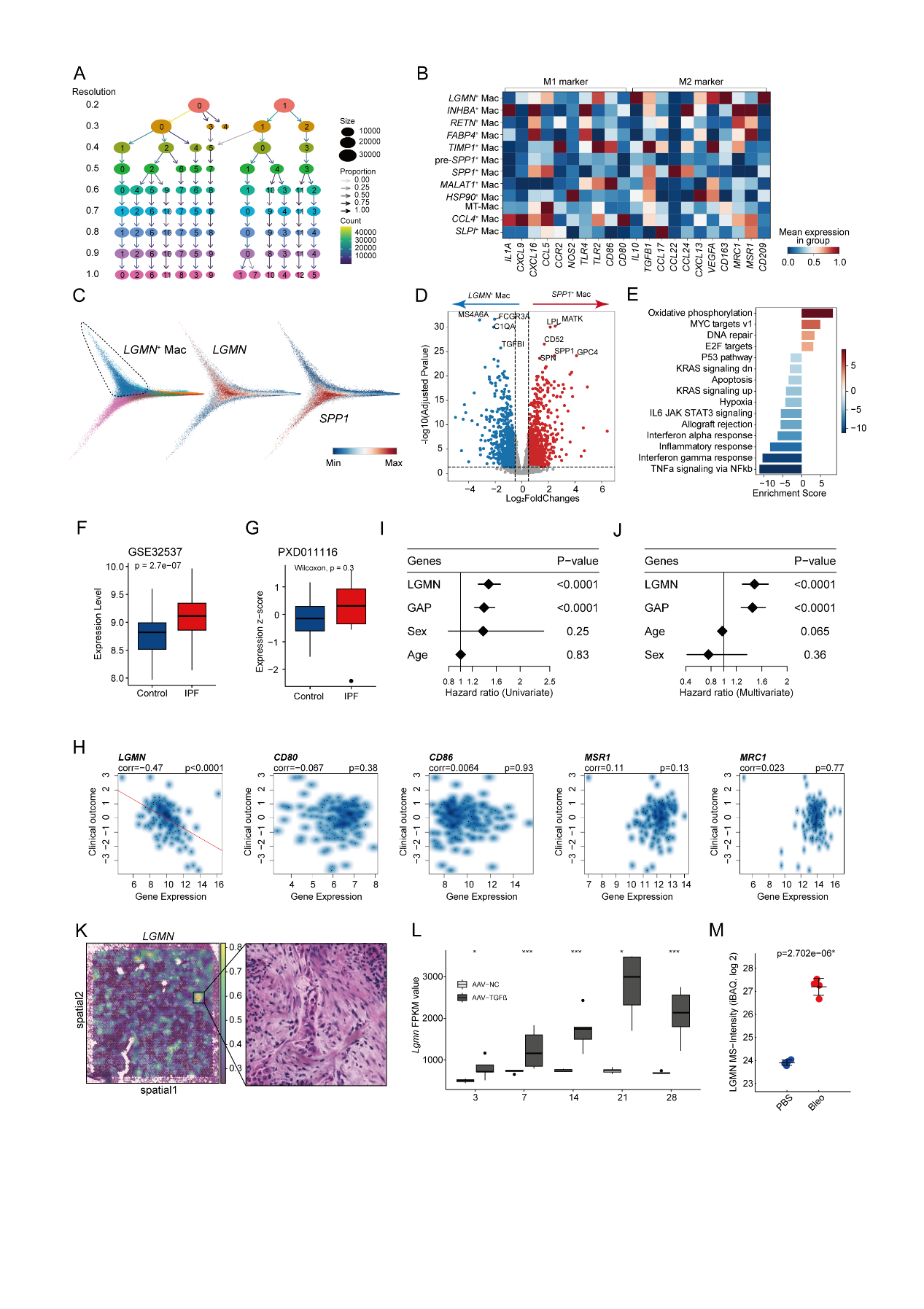


Fig. S2.

(A) Leiden clustering tree depicting the hierarchical organization of lung immune cell populations across resolution levels. (B) Heatmap of canonical M1- and M2-associated gene expression across macrophage subsets. (C) Diffmap visualization of LGMN and SPP1 expression gradients across all macrophage populations; color indicates cell density (blue: low, red: high). (D) Volcano plot of differentially expressed genes between *LGMN*⁺ (blue) and *SPP1*⁺ (red) macrophages (pseudo-bulk RNA-seq). (E) Bar plot of GSEA results for HALLMARK gene sets; red denotes pathways significantly upregulated in *SPP1*⁺ macrophages, blue in *LGMN*⁺ macrophages. (F) Boxplot of *LGMN* mRNA expression in IPF (GSE32537) versus controls. (G) Boxplot of LGMN protein expression in IPF (PXD011116) versus controls. (H) Scatter plots showing correlations between *LGMN, CD80, CD86, MSR1,* and *MRC1* expression and clinical outcomes in IPF. (I) Univariate Cox regression forest plot: HRs for LGMN, GAP, sex, and age; HR > 1 indicates increased risk. (J) Multivariate Cox regression forest plot adjusted for age and sex. (K) Spatial transcriptomic map of LGMN expression (left) overlaid on H&E-stained lung histology (right). (L) Longitudinal quantification of Lgmn expression in mouse lungs following TGFβ-induced fibrosis (days 0–21). (M) Quantification of LGMN protein intensity in lung tissue from PBS- and BLM-treated mice.


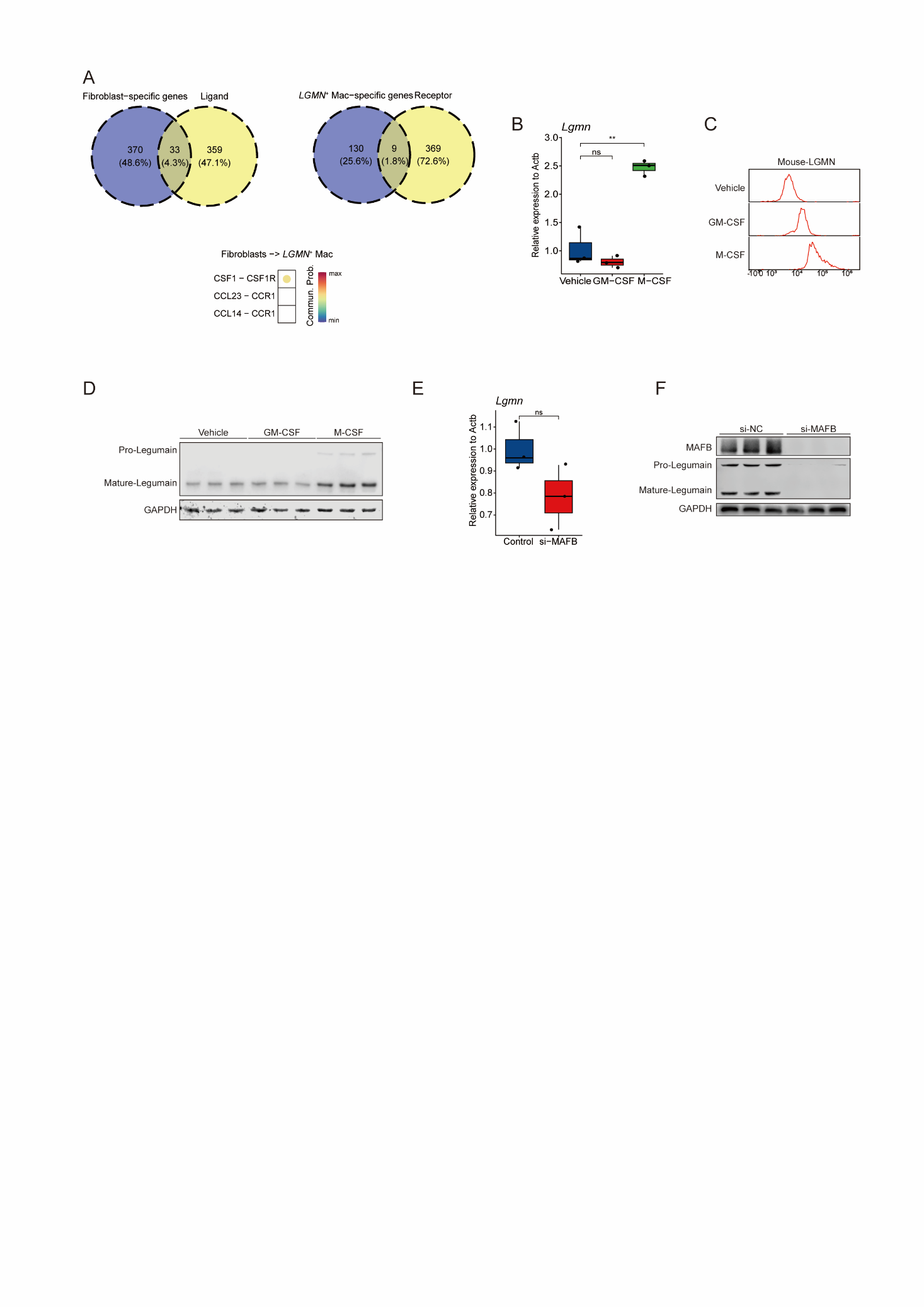


Fig. S3.

(A) Venn diagrams showing overlap between ligand-receptor pairs expressed by fibroblasts and LGMN⁺ macrophages. Heatmap below indicates common ligand-receptor interactions with predicted probability scores (range: min to max), emphasizing CSF1–CSF1R as a key axis in fibroblast-to-macrophage crosstalk. (B) Quantitative RT-PCR analysis of *Lgmn* mRNA expression in bone marrow-derived macrophages (BMDMs) treated with vehicle, GM-CSF, or M-CSF. *Lgmn* expression is significantly upregulated by M-CSF (p < 0.01), while GM-CSF has no significant effect. Data shown as mean ± SEM (n = 3 per group). (C) Flow cytometry histogram of mouse LGMN protein expression in BMDMs after treatment with vehicle, GM-CSF, or M-CSF. M-CSF induces robust surface expression of LGMN, whereas GM-CSF shows minimal effect. (D) Western blot analysis of pro- and mature LGMN protein levels in BMDMs under the same conditions. M-CSF enhances both forms of LGMN, consistent with increased expression and processing. β-actin serves as loading control. (E) qRT-PCR quantification of Lgmn expression in BMDMs transfected with siRNA targeting MAFB (si-MAFB) versus control siRNA (si-NC). Knockdown of *MAFB* significantly reduces *Lgmn* expression (p < 0.05; n = 3). (F) Western blot confirming *MAFB* knockdown efficiency and its impact on LGMN protein level. MAFB silencing leads to reduced expression of both pro- and mature LGMN. β-actin used as loading control.


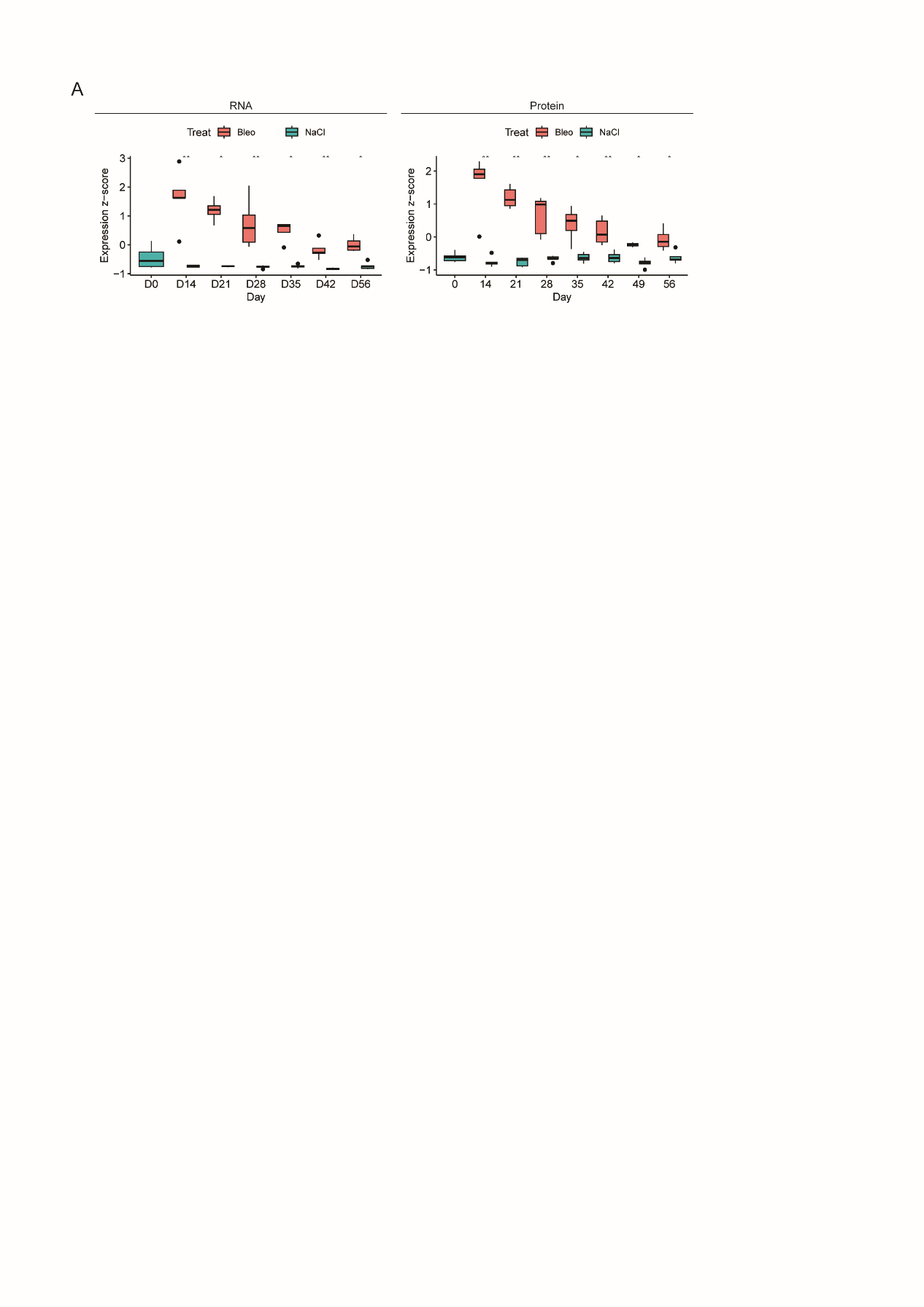


Fig. S4

(A) Longitudinal analysis of Lgmn expression across time points following intratracheal BLM or NaCl administration. Left panel: RNA-level expression (z-score normalized). Right panel: Protein-level expression (z-score normalized). Data shown as boxplots (median ± interquartile range; individual data points shown), with statistical significance determined by two-way ANOVA followed by Tukey’s post hoc test (**p < 0.01, ***p < 0.001).


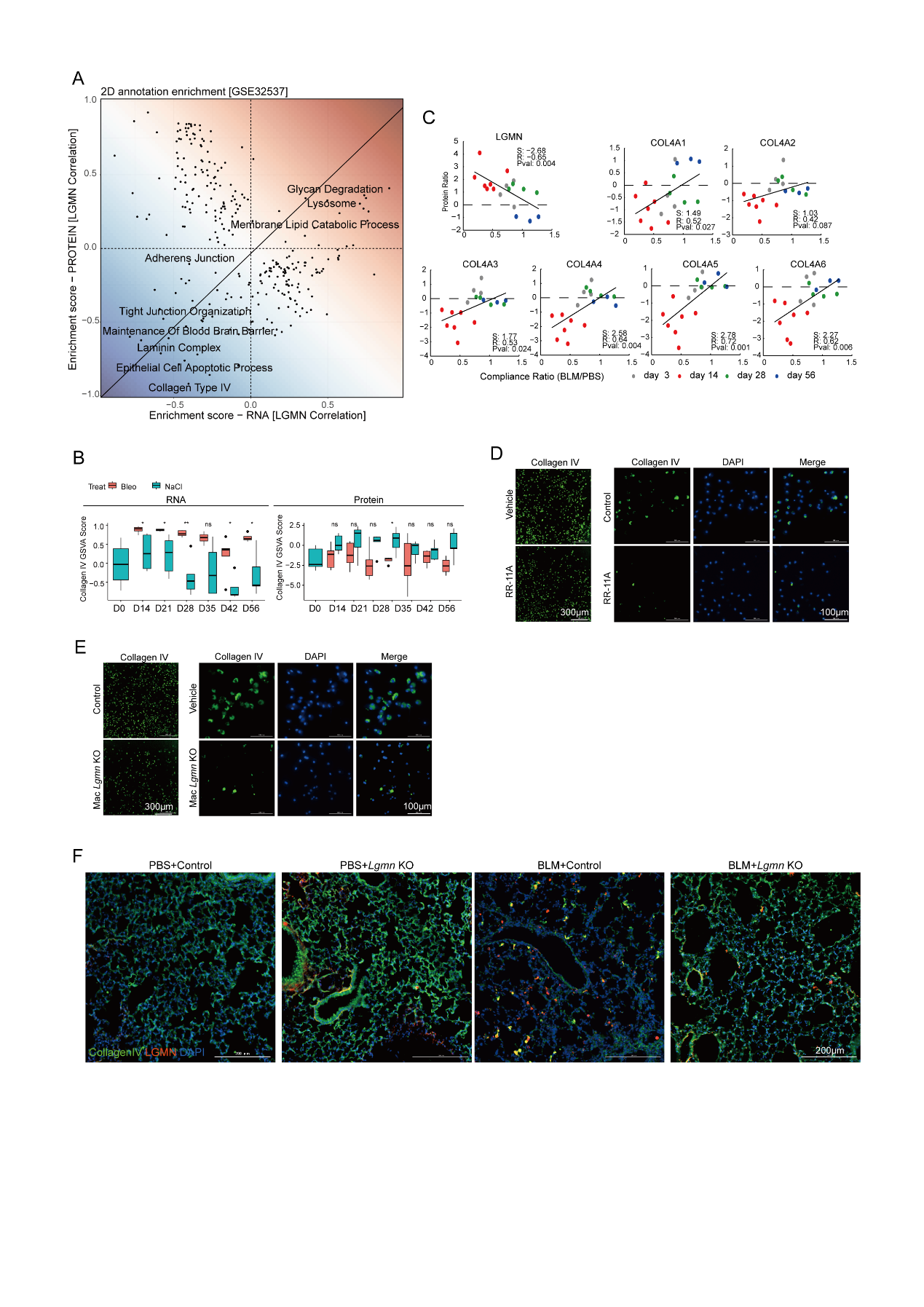


Fig. S5.

(A) Two-dimensional annotation enrichment plot showing gene ontology (GO) terms enriched in genes correlated with LGMN expression (GSE32537). (B) Boxplots showing Collagen IV gene expression (z-score normalized) at RNA and protein levels over time after BLM or NaCl treatment. (C) Scatter plots showing correlation between protein levels of LGMN and COL4A1–COL4A6 isoforms and lung compliance ratio (BI.M/PD15) across time points post- BLM challenge. (D) Immunofluorescence staining of Collagen IV (green) in mouse lungs treated with vehicle or RR-11A. Scale bars: 300 µm (top), 100 µm (bottom). (E) Immunofluorescence staining of Collagen IV in control and macrophage-specific Lgmn KO mice. Lgmn deficiency leads to reduced Collagen IV accumulation compared to controls, particularly in perivascular and alveolar regions. Scale bars: 300 µm (left), 100 µm (right). (F) Immunofluorescence of Collagen IV (green), LGMN (red), and DAPI (blue) in lungs from PBS- or BLM-treated wild-type and Lgmn KO mice. Scale bar: 200 µm.


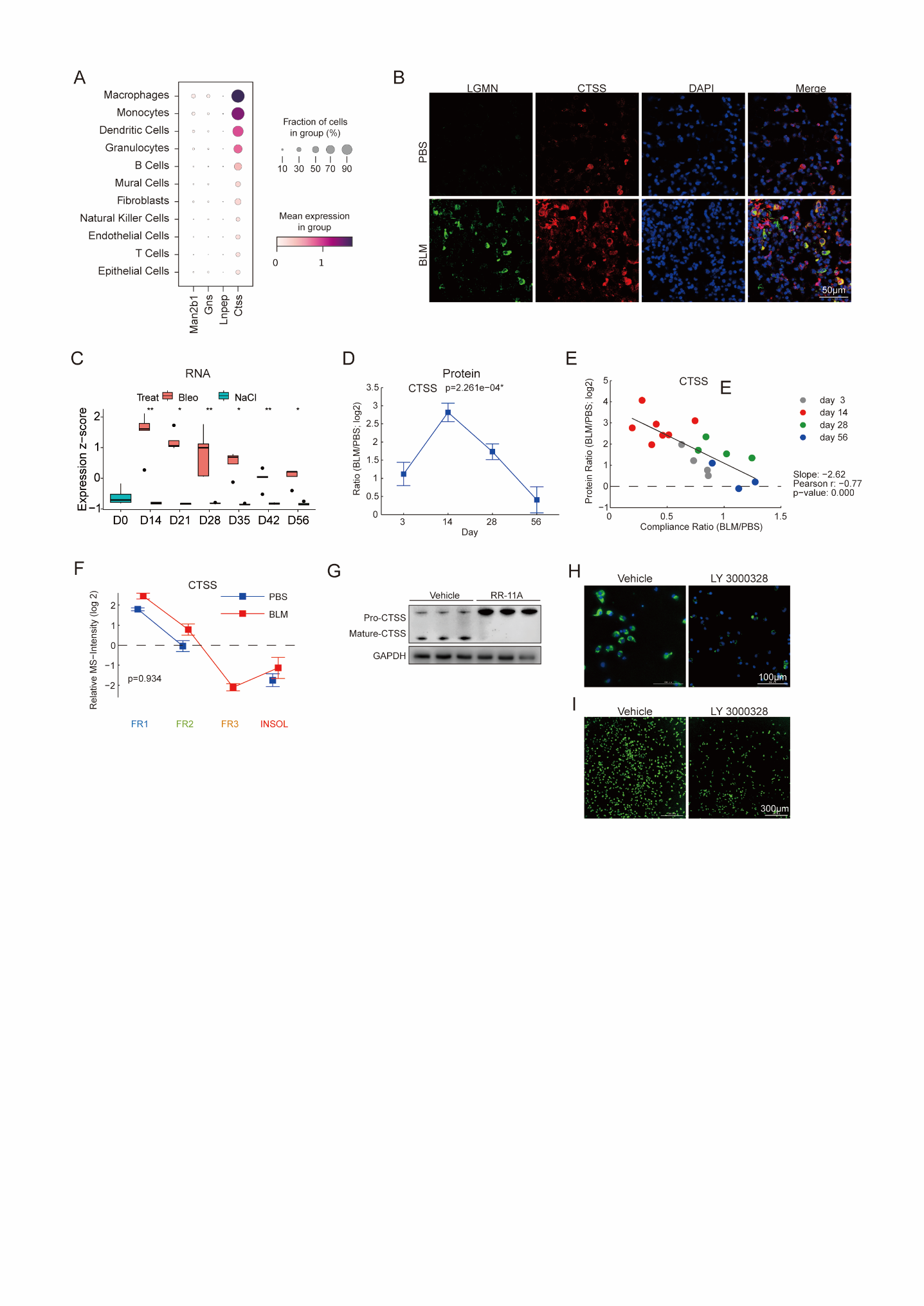


Fig. S6.

(A) Heatmap showing expression profiles of Ctss across cell types in mouse lung tissue from BLM-treated mice. CTSS is predominantly expressed in macrophages and monocytes. Cell-type proportions and mean expression levels are indicated. (B) Immunofluorescence staining of LGMN (green), CTSS (red), and DAPI (blue) in lungs from PBS- and BLM-treated mice. In BLM-treated mice, CTSS+ cells accumulate in alveolar spaces and interstitial regions, co-localizing with LGMN+ macrophages. Scale bar: 50 µm. (C) Longitudinal analysis of Ctss mRNA expression (z-score normalized) over time after BLM or NaCl administration. Expression increases significantly by day 14, peaks at day 21, and declines toward baseline by day 56 (n = 4–6 per group; **p < 0.01). (D) Quantification of CTSS protein levels (log₂ ratio BLM/PBS) over time. Protein levels peak at day 14 and decline by day 56 (p = 2.26×10⁻⁰⁴). (E) Scatter plot showing inverse correlation between CTSS protein levels and lung compliance ratio (BLM/PBS). Higher CTSS expression correlates with reduced lung compliance (slope = −2.82, Pearson r = −0.77, p = 0.000), indicating its role in mechanical dysfunction. (F) Relative mass spectrometry intensity of CTSS isoforms (FR1, FR2, FR3, INSOL) in PBS- and BLM-treated lungs. CTSS undergoes dynamic processing, with FR1 and FR2 increasing early, followed by accumulation of insoluble forms (INSOL) at later time points (p = 0.934, no significant difference between groups). (G) Western blot analysis of pro- and mature CTSS protein levels in BMDMs after treatment with vehicle or RR-11A. (H-I) Immunofluorescence of DQ-Collagen IV in PBMC-derived macrophages treated with vehicle or LY3000328 LY3000328 significantly reduces CTSS signal intensity in alveolar and interstitial compartments.
