# Supplementary figures and images for: Monocyte-Derived LGMN+ Macrophages Divert Lung Injury Outcomes toward Fibrosis through Matrix Remodeling
Source: Research (Wash D C). 2026 Jun 29;9:1341. doi: 10.34133/research.1341 (PMC13311260; doi:10.34133/research.1341)

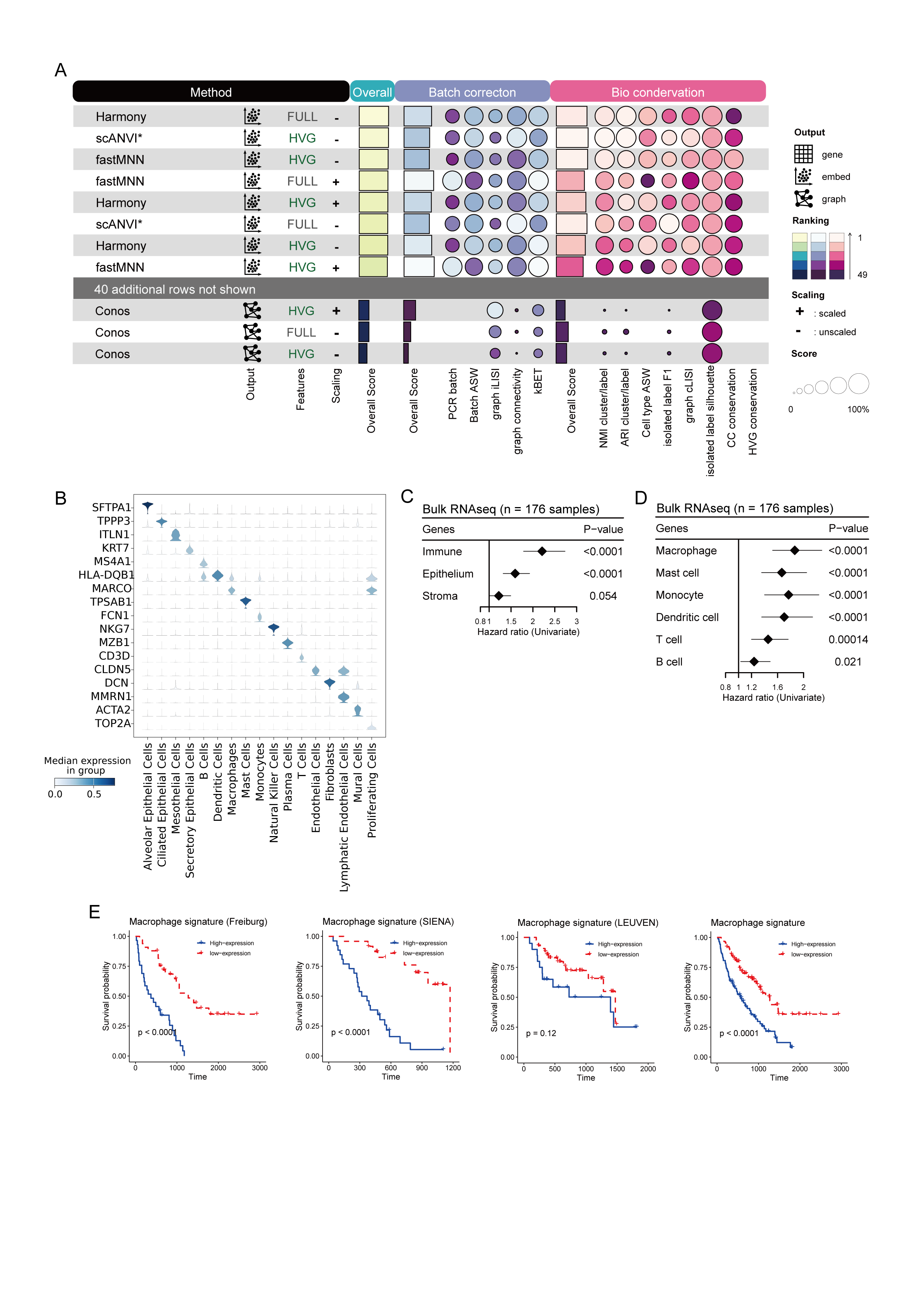

Supplement: Supplementary 1 — Figs. S1 to S6 Tables S1 to S5 [file research.1341.f1.zip › FigureS1.tif]

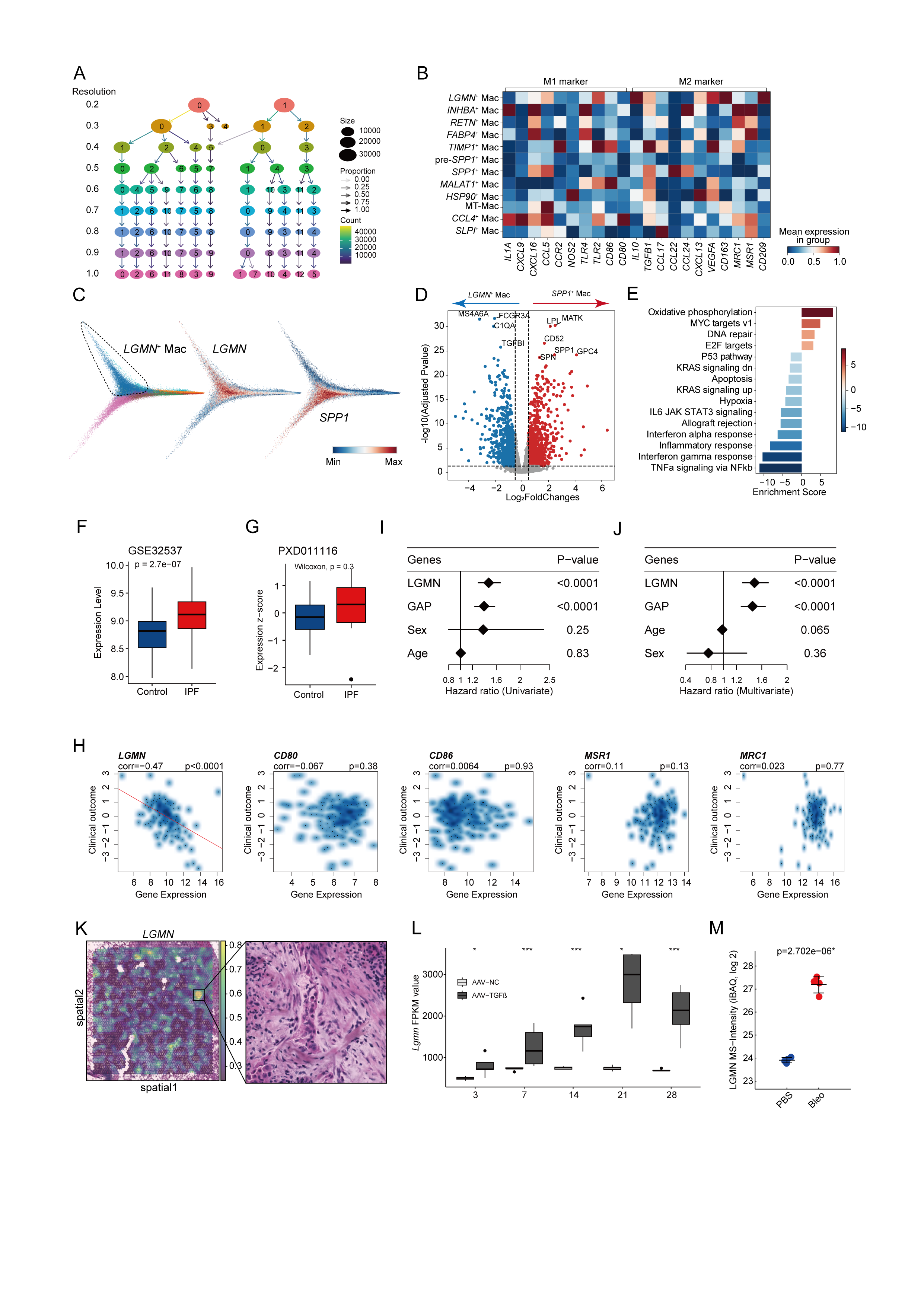

Supplement: Supplementary 1 — Figs. S1 to S6 Tables S1 to S5 [file research.1341.f1.zip › FigureS2.tif]

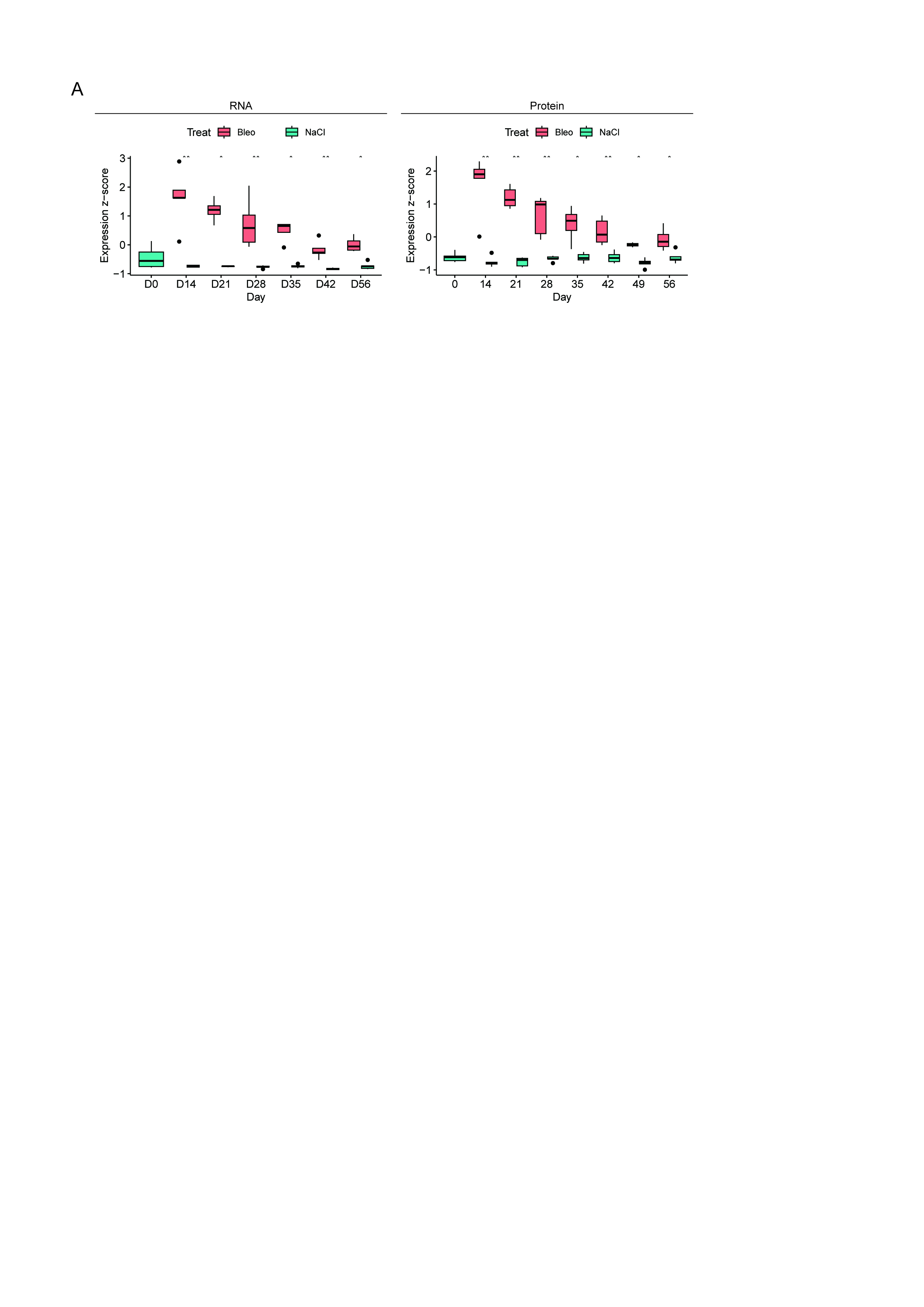

Supplement: Supplementary 1 — Figs. S1 to S6 Tables S1 to S5 [file research.1341.f1.zip › FigureS3.tif]

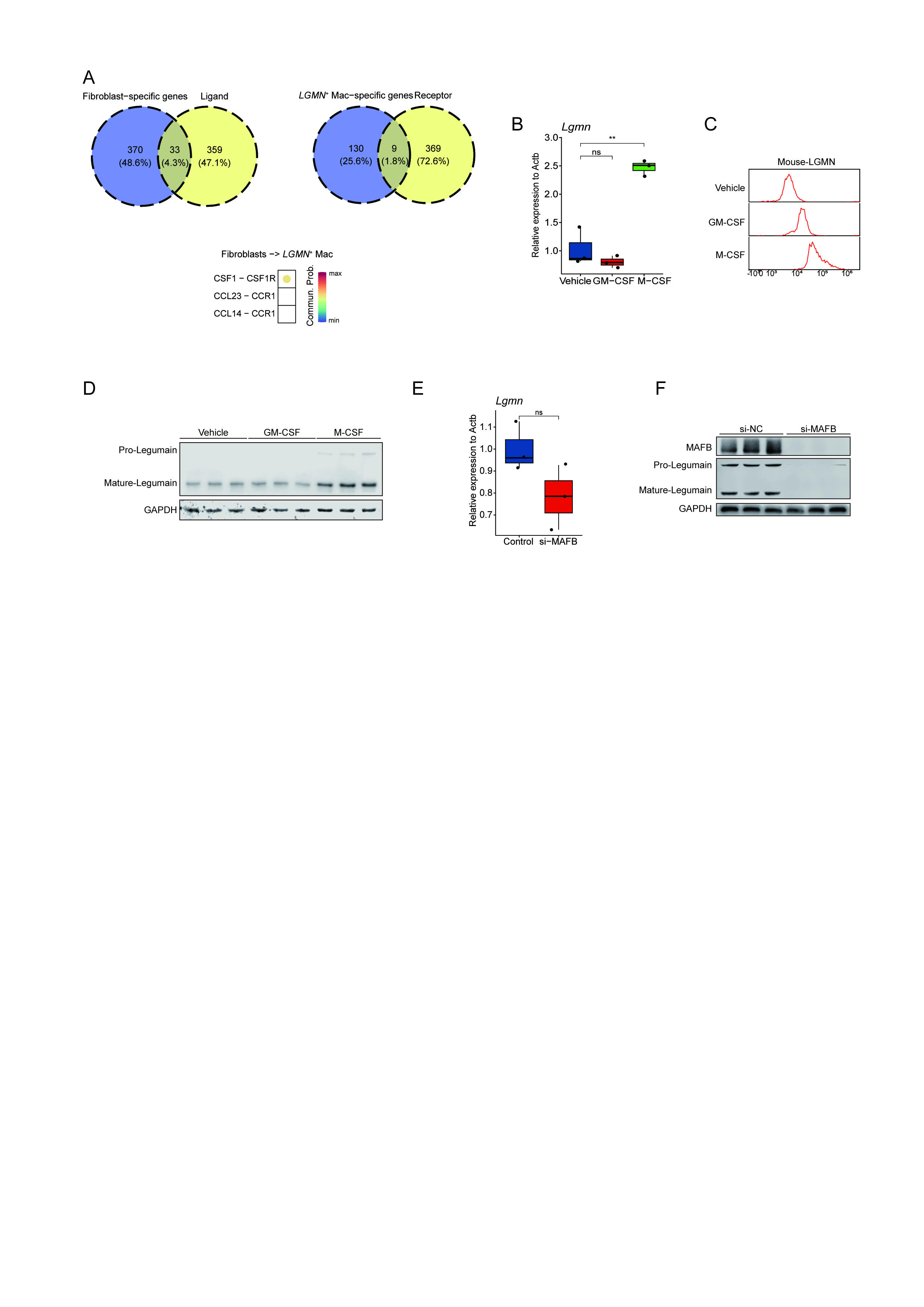

Supplement: Supplementary 1 — Figs. S1 to S6 Tables S1 to S5 [file research.1341.f1.zip › FigureS4.tif]

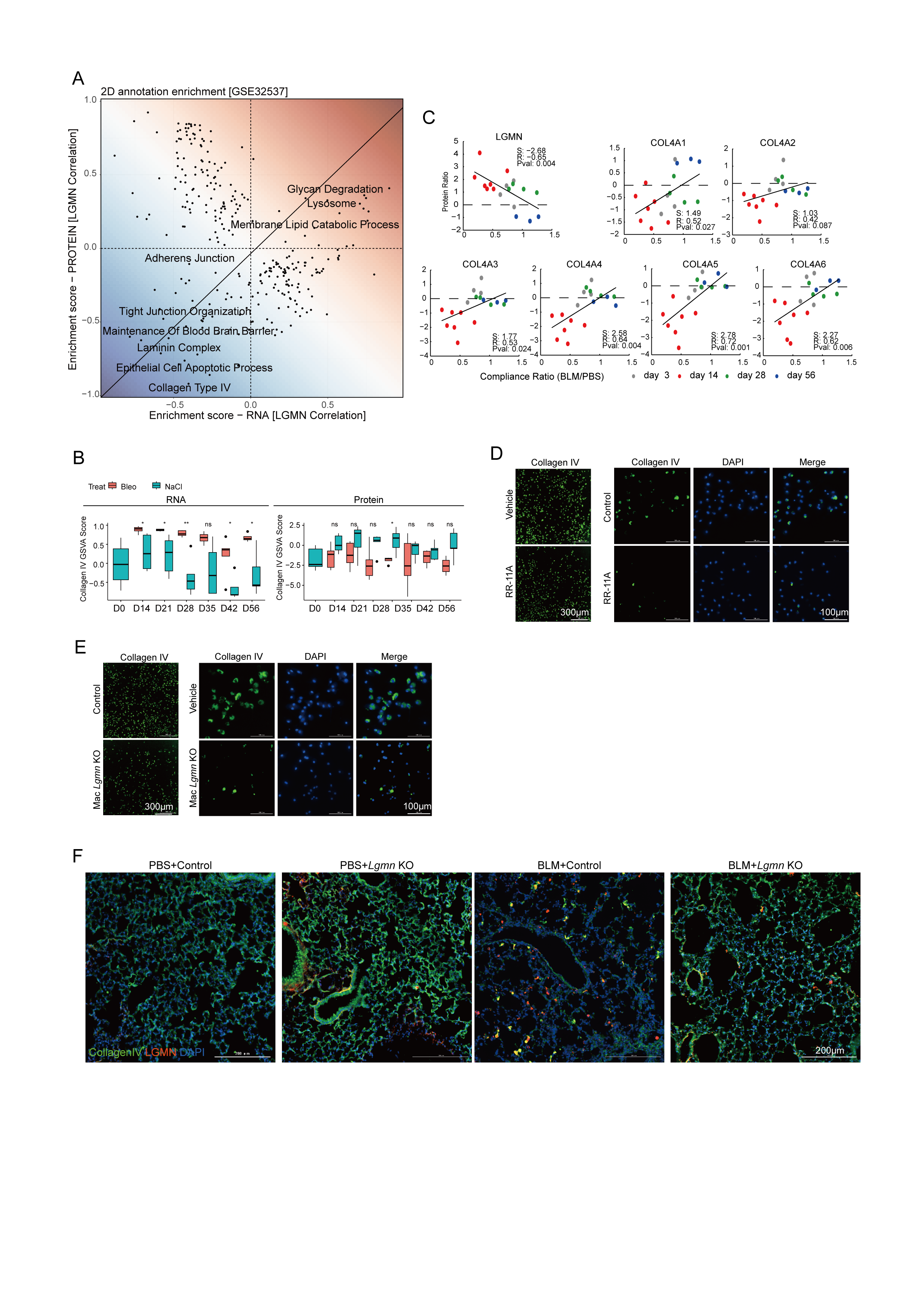

Supplement: Supplementary 1 — Figs. S1 to S6 Tables S1 to S5 [file research.1341.f1.zip › FigureS5.tif]

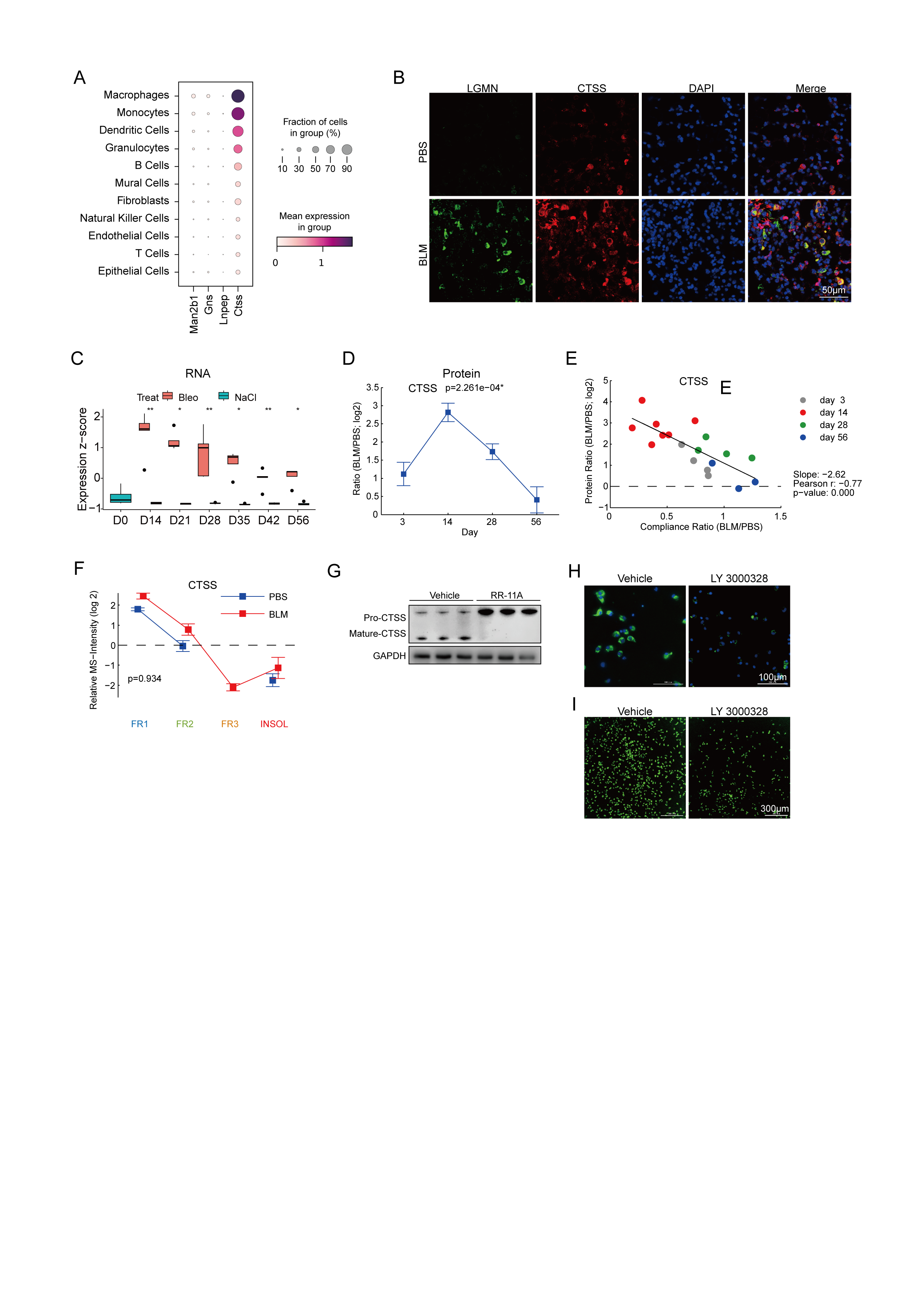

Supplement: Supplementary 1 — Figs. S1 to S6 Tables S1 to S5 [file research.1341.f1.zip › FigureS6.tif]
